# Supplementary material for: Maternal anaemia during early pregnancy and the risk of neonatal outcomes: a prospective cohort study in Central China
Source: BMJ Paediatr Open. 2024 Jan 17;8(1):e001931. doi: 10.1136/bmjpo-2023-001931 (PMC10806529; doi:10.1136/bmjpo-2023-001931)
Supplement: Supplementary data [file bmjpo-2023-001931supp001.pdf]

Supplement Table 1 Adjusted risk ratios for covariates in Model A and B associated with neonatal adverse outcomes

Covariates	Model A[b]				Model B [c]			
	Preterm birth	Low birth weight	SGA	Congenital malformation	Preterm birth	Low birth weight	SGA	Congenital malformation
Maternal age (years)								
<35	Reference	Reference	Reference	Reference	Reference	Reference	Reference	Reference
≥35	1.07(0.99-1.16)	1.37(1.23-1.51) *	1.17(1.06-1.28) *	1.11(0.95-1.29) *	1.07(0.99-1.16)	1.36(1.23-1.52) *	1.16(1.06-1.28) *	1.11(0.96-1.29) *
Maternal BMI								
18.5-23.9	Reference	Reference	Reference	Reference	Reference	Reference	Reference	Reference
<18.5	2.08(1.20-3.60) *	4.19(2.34-7.49) *	2.35(1.30-4.27) *	-	2.04(1.18-3.53) *	3.97(2.21-7.13) *	2.29(1.26-4.15) *	-
24-27.9	0.79(0.74-0.85) *	0.51(0.46-0.56) *	0.73(0.67-0.79) *	1.17(1.00-1.37) *	0.79(0.74-0.85) *	0.50(0.45-0.55) *	0.73(0.67-0.79) *	1.17(1.01-1.37) *
≥28	0.75(0.69-0.81) *	0.46(0.41-0.51) *	0.65(0.59-0.72) *	1.43(1.22-1.51) *	0.75(0.70-0.82) *	0.46(0.41-0.51) *	0.66(0.60-0.73) *	1.43(1.21-1.67) *
Residence								
Urban	Reference	Reference	Reference	Reference	Reference	Reference	Reference	Reference
Rural	1.22(1.16-1.29) *	1.97(1.82-2.13) *	1.40(1.30-1.50) *	1.36(1.22-1.51) *	1.23(1.16-1.29) *	2.01(1.85-2.17) *	1.40(1.31-1.51) *	1.32(1.19-1.47) *
Education level								
Junior high school or below	Reference	Reference	Reference	Reference	Reference	Reference	Reference	Reference
Senior middle school	0.74(0.66-0.84) *	0.60(0.61-0.70) *	0.70(0.61-0.81) *	0.89(0.68-1.15)	0.74(0.66-0.84) *	0.59(0.51-0.69) *	0.70(0.61-0.81) *	0.87(0.67-1.14)
College	0.61(0.56-0.67) *	0.34(0.30-0.38) *	0.52(0.47-0.58) *	0.78(0.65-0.95) *	0.61(0.56-0.67) *	0.33(0.29-0.37) *	0.52(0.47-0.58) *	0.78(0.64-0.95) *
Master or above	0.53(0.47-0.60) *	0.20(0.17-0.24) *	0.40(0.35-0.47) *	0.89(0.70-1.12)	0.53(0.47-0.59) *	0.19(0.16-0.24) *	0.41(0.34-0.47) *	0.87(0.69-1.11)
Ethnicity								
Han	Reference	Reference	Reference	Reference	Reference	Reference	Reference	Reference

Minority	0.83(0.66-1.04)	0.81(0.58-1.13)	0.86(0.65-1.14)	0.72(0.48-1.06)	0.82(0.66-1.03)	0.78(0.56-1.10)	0.85(0.63-1.13)	0.72(0.49-1.08)
Parity								
0	Reference	Reference	Reference	Reference	Reference	Reference	Reference	Reference
1-3	1.01(0.95-1.08)	1.17(1.06-1.29) *	1.04(0.96-1.13)	0.84(0.75-0.95) *	1.01(0.95-1.08)	1.18(1.07-1.29) *	1.04(0.96-1.12)	0.85(0.75-0.95) *
≥4	1.09(0.98-1.21)	1.43(1.24-1.65) *	1.13(0.99-1.29)	0.73(0.59-0.91) *	1.09(0.98-1.21)	1.41(1.22-1.64) *	1.13(0.98-1.28)	0.73(0.59-0.91) *
Per caput monthly family income (RMB)								
≤2500	Reference	Reference	Reference	Reference	Reference	Reference	Reference	Reference
2500-5000	1.08(1.00-1.16) *	1.27(1.13-1.42) *	1.17(1.06-1.28) *	0.84(0.73-0.97) *	1.09(1.01-1.18) *	1.33(1.18-1.49) *	1.19(1.08-1.31) *	0.86(0.74-0.99) *
>5000	0.95(0.87-1.03)	0.92(0.81-1.05)	0.98(0.89-1.10)	0.88(0.75-1.02)	0.96(0.88-1.04)	0.94(0.83-1.08)	0.99(0.89-1.11)	0.92(0.78-1.05)
Folic acid use *								
Yes	Reference	Reference	Reference	Reference	Reference	Reference	Reference	Reference
No	1.21(1.07-1.36) *	1.76(1.52-2.05) *	1.35(1.17-1.56) *	1.57(1.28-1.94) *	1.21(1.07-1.36) *	1.78(1.53-2.08) *	1.35(1.17-1.56) *	1.58(1.28-1.95) *
Gestational diabetes mellitus								
Yes	-	-	-	-	Reference	Reference	Reference	Reference
No	-	-	-	-	0.97(0.91-1.05)	1.10(0.99-1.23)	1.02(0.93-1.11)	0.87(0.75-1.02)
Gestational hypertension								
Yes	-	-	-	-	Reference	Reference	Reference	Reference
No	-	-	-	-	1.82(1.60-2.06) *	3.95(3.42-4.56) *	2.26(1.97-2.61) *	0.88(0.65-1.19)
Hyperlipidemia								
Yes	-	-	-	-	Reference	Reference	Reference	Reference
No	-	-	-	-	1.05(0.82-1.35)	1.06(0.74-1.53）	1.14(0.84-1.55)	0.86(0.50-1.47)

Hyperthyroidism								
Yes	-	-	-	-	Reference	Reference	Reference	Reference
No	-	-	-	-	0.97(0.77-1.18)	0.46(0.32-0.67) *	0.99(0.78-1.27)	0.86(0.57-1.30)
Placenta previa								
Yes	-	-	-	-	Reference	Reference	Reference	Reference
No	-	-	-	-	0.81(0.65-1.01)	0.53(0.37-0.76) *	0.67(0.50-0.89) *	3.91(3.09-4.93) *

SGA= small for gestational age.

[b] Model A adjusted for maternal age, maternal BMI, residence, education level, ethnicity, parity, folate use, and per caput monthly family income.

[c] Model B adjusted for all maternal complications during pregnancy in addition to covariates in model A.

* $P < 0.005$.
