# Supplementary material for: Maternal anaemia during early pregnancy and the risk of neonatal outcomes: a prospective cohort study in Central China
Source: BMJ Paediatr Open. 2024 Jan 17;8(1):e001931. doi: 10.1136/bmjpo-2023-001931 (PMC10806529; doi:10.1136/bmjpo-2023-001931)
Supplement: Author's manuscript [file bmjpo-2023-001931.draft_revisions.pdf]

BMJ Paediatrics Open

BMJ Paediatrics Open is committed to open peer review. As part of this commitment we make the peer review history of every article we publish publicly available.

When an article is published we post the peer reviewers' comments and the authors' responses online. We also post the versions of the paper that were used during peer review. These are the versions that the peer review comments apply to.

The versions of the paper that follow are the versions that were submitted during the peer review process. They are not the versions of record or the final published versions. They should not be cited or distributed as the published version of this manuscript.

BMJ Paediatrics Open is an open access journal and the full, final, typeset and author-corrected version of record of the manuscript is available on our site with no access controls, subscription charges or pay-per-view fees (http://bmjpaedsopen.bmj.com).

If you have any questions on BMJ Paediatrics Open's open peer review process please email info.bmjpo@bmj.com

BMJ Paediatrics Open

Maternal anemia during early pregnancy and the risk of neonatal outcomes: a prospective cohort study in Central China

Journal:	*BMJ Paediatrics Open*
Manuscript ID	bmjpo-2023-001931
Article Type:	Original research
Date Submitted by the Author:	23-Feb-2023
Complete List of Authors:	Liu, Yiping; Central South University, Department of Epidemiology and Health Statistics<br>Zhong, Taowei; Central South University, Department of Epidemiology and Health Statistics<br>Song, Xinli; Central South University, Department of Epidemiology and Health Statistics<br>Zhang, Senmao; Central South University, Department of Epidemiology and Health Statistics<br>Sun, Mengting; Central South University, Department of Epidemiology and Health Statistics<br>Liu, Xiaoying; Changsha Medical University, Public Health Institute; The Hospital of Trade-Business in Hunan Province<br>Wei, Jianhui; Central South University, Department of Epidemiology and Health Statistics<br>Shu, Jing; Central South University, Department of Epidemiology and Health Statistics<br>Yang, Tubao; Central South University, Department of Epidemiology and Health Statistics; Hunan Provincial Key Laboratory of Clinical Epidemiology<br>Wang, Tingting; Central South University, Department of Epidemiology and Health Statistics; Hunan Provincial Maternal and Child Health Care Hospital<br>Qin, Jiabi; Central South University, Department of Epidemiology and Health Statistics; Hunan Provincial Maternal and Child Health Care Hospital
Keywords:	Epidemiology, Growth, Neonatology

SCHOLARONE™
Manuscripts

BMJ

I, the Submitting Author has the right to grant and does grant on behalf of all authors of the Work (as defined in the below author licence), an exclusive licence and/or a non-exclusive licence for contributions from authors who are: i) UK Crown employees; ii) where BMJ has agreed a CC-BY licence shall apply, and/or iii) in accordance with the terms applicable for US Federal Government officers or employees acting as part of their official duties; on a worldwide, perpetual, irrevocable, royalty-free basis to BMJ Publishing Group Ltd ("BMJ") its licensees and where the relevant Journal is co-owned by BMJ to the co-owners of the Journal, to publish the Work in this journal and any other BMJ products and to exploit all rights, as set out in our licence.

The Submitting Author accepts and understands that any supply made under these terms is made by BMJ to the Submitting Author unless you are acting as an employee on behalf of your employer or a postgraduate student of an affiliated institution which is paying any applicable article publishing charge ("APC") for Open Access articles. Where the Submitting Author wishes to make the Work available on an Open Access basis (and intends to pay the relevant APC), the terms of reuse of such Open Access shall be governed by a Creative Commons licence – details of these licences and which Creative Commons licence will apply to this Work are set out in our licence referred to above.

Other than as permitted in any relevant BMJ Author's Self Archiving Policies, I confirm this Work has not been accepted for publication elsewhere, is not being considered for publication elsewhere and does not duplicate material already published. I confirm all authors consent to publication of this Work and authorise the granting of this licence.

Confidential: For Review Only

Maternal anemia during early pregnancy and the risk of neonatal outcomes: a prospective cohort study in Central China

Yiping Liu[1†], Taowei Zhong[1†], Xinli Song[1], Senmao Zhang[1], Mengting Sun[1], Xiaoying Liu[2, 3], Jianhui Wei[1], Jing Shu[1], Tubao Yang[1, 4*], Tingting Wang[1*], Jiabi Qin[1, 4, 5, 6*]

[1] Department of Epidemiology and Health Statistics, Xiangya School of Public Health, Central South University, Changsha, Hunan, China

[2] Public Health Institute, Changsha Medical University, Changsha, Hunan, China

[3] The Hospital of Trade-Business in Hunan Province, Changsha, Hunan, China

[4] Hunan Provincial Key Laboratory of Clinical Epidemiology, Changsha, Hunan, China

[5] NHC Key Laboratory of Birth Defect for Research and Prevention, Hunan Provincial Maternal and Child Health Care Hospital, Changsha, Hunan, China

[6] Guangdong Cardiovascular Institute, Guangdong Provincial People's Hospital, Guangdong Academy of Medical Sciences, Guangzhou, Guangdong, China

† Co-First Author: Yiping Liu, Taowei Zhong

*** Co-Corresponding Author**

Tubao Yang, Department of Epidemiology and Health Statistics, Xiangya School of Public Health, Central South University, 110 Xiangya Road, Changsha, Hunan 410078, China;

Telephone: (+86)13973132376; Email: 1064960669@qq.com;

Tingting Wang, Department of Epidemiology and Health Statistics, Xiangya School of Public Health, Central South University, 110 Xiangya Road, Changsha, Hunan 410078, China;

Telephone: (+86)15273188582; E-mail: wangting91123@126.com;

Jiabi Qin, Department of Epidemiology and Health Statistics, Xiangya School of Public Health, Central South University, 110 Xiangya Road, Changsha, Hunan 410078, China;

Telephone: (+86)15974269544; E-mail: qinjiabi123@163.com.

Word count:2605 words

Confidential: For Review Only

Abstract

Background: The purpose of this study was to explore the association between anemia during early pregnancy and the risk of neonatal outcomes.

Methods: We collected clinical data from pregnant females (≥18 years) who received their first antenatal care between 8-14 weeks of gestation in Hunan provincial Maternal and Child Health Care Hospital. Mantel-Haenszel Chi-square test was utilized for trends for neonatal outcomes. Multivariate logistic regression model was used to analyze the association between anemia during early pregnancy and the risk of neonatal outcomes. In addition, sensitivity analysis was further performed to assess the robustness of the results.

Results: The prospective cohort study ultimately included 34,08 singleton pregnancies. In this study, the rate of anemia during early pregnancy was 16.3%. Our data showed that there may be an upward curve relationship between the rate of preterm birth, low birth weight as well as SGA and the severity of maternal anemia ($P<0.05$). After adjustment, the risk estimates of preterm birth (mild anemia aOR 1.61 (95% CI 1.39–1.85), moderate anemia aOR 1.62 (95% CI 1.39–1.88), and severe anemia aOR 1.39 (95% CI 1.17–1.63), respectively), low birth weight (mild anemia aOR 1.61 (95% CI 1.39–1.85), moderate anemia aOR 1.62 (95% CI 1.39–1.88), and severe anemia aOR 1.39 (95% CI 1.17–1.63), respectively) and SGA (mild anemia aOR 1.61 (95% CI 1.39–1.85), moderate anemia aOR 1.62 (95% CI 1.39–1.88), and severe anemia aOR 1.39 (95% CI 1.17–1.63), respectively) significantly increased in offspring of pregnant females who had anemia. However, the association between anemia during early pregnancy and the risk of congenital malformation was not observed. Sensitivity analysis verified the stability of the results.

Conclusions: Maternal anemia during early pregnancy was associated with an increased risk of preterm birth, low birth weight and SGA, and their rates may increase with the severity of maternal anemia.

Trial registration: This study was registered in the Chinese Clinical Trial Registry (Registration number: ChiCTR1800016635).

Keywords: Epidemiology, Growth, Neonatology

Confidential: For Review Only

What is known about the subject

- Anemia is the most common nutritional deficiency in pregnancy across the world.
- Maternal anemia is related to adverse neonatal outcomes, but there are inconsistent findings.
- These associations of maternal anemia with adverse neonatal outcomes may vary when the severity of gestational anemia is considered.

What this study adds:

- Maternal anemia in the first trimester (8-14 weeks) is associated with an increased risk of preterm birth, low birth weight and small for gestational age (SGA).
- The more severe the maternal anemia, the higher the incidence of preterm birth, low birth weight and SGA.

How this study may affect research, practice or policy

- This study may ensure the reliability of the relation between maternal anemia and adverse neonatal outcomes.
- To involve pregnant women with different severity of anemia in timely prenatal care may be beneficial to reducing the risk of adverse neonatal outcomes.

Confidential: For Review Only

INTRODUCTION

Anemia is a pathophysiological condition in which the level of hemoglobin (Hb) and/or the quantity of red blood cells are lower than established threshold values, thereby impairing the capacity of the oxygen-carrying to tissues.[1] It has been claimed to be the most common nutritional deficiency in pregnancy across the world, bringing high burden to developed and developing countries.[2, 3] Anemia in pregnancy constitutes a global public health challenge with its high prevalence. Stevens *et al.*[4] reported that the global prevalence of anemia in pregnant women was 38%, affecting approximately 32 million individuals. The significance of reducing and properly managing maternal anemia has been recognized globally. Current targets which set in Sustainable Development Goal 3 and Global Nutrition Targets 2025 call for a 50% decrease of anemia in women of reproductive age by the year 2025.[5]

Anemia during pregnancy is a key contributor to adverse neonatal outcomes.[6] Findings have widely demonstrated associations between maternal anemia and adverse neonatal outcomes, such as low birth weight, preterm birth, and small for gestational age (SGA).[7, 8] But these associations may be different when the severity of gestational anemia is taken into account. Limited data suggested that risks of low birth weight decreased in offspring of pregnant women who have mild anemia compared with pregnant women who have no anemia.[9] A study of 421 pregnant women in India indicated that an association between severity of anemia and low birth weight as well as SGA.[10]

The Hb concentration of pregnant women decreases from the second trimester.[11] This is due to the reason that expansion of plasma volume begins in the second trimester of pregnancy physiologically.[12] Therefore, measurements of Hb made during or after the second trimester are likely to overvalue the prevalence of anemia. The level of Hb estimated before the 14th week of gestation may reflect Hb levels in pre-pregnancy women and guide future timing and delivery of maternal interventions and prenatal care.[13] So, we took the maternal anemia in the first trimester for the analysis in this study.

To sum up, this study was based on a prospective cohort study to further clarify the association between the severity of anemia during early pregnancy and the risk of adverse neonatal outcomes in China.

METHODS

Recruitment of study participants

A prospective cohort study design was implemented. From March 13, 2013 to January 31, 2020, pregnant females (≥18 years) who received their first antenatal care at 8-14 weeks of gestation with the intention of continuously receiving prenatal care throughout gestation at Hunan provincial Maternal and Child Health Care Hospital, in Hunan Province, China were approached and recruited into this cohort. Gestational weeks were based on the last menstrual period data, or estimated by ultrasonography if menstruation was irregular. During these pregnant women's first antenatal visit, a total of 49,456 pregnant females met these inclusion criteria were invited to involve in this present cohort. Considering assisted reproductive technology (ART) may lead to misestimation of the prevalence of preterm birth and low birth weight, woman concepted with the help of ART were not included in our study. We excluded 15,369 women on the strength of the following exclusion criteria: termination of pregnancy by artificial abortion or induced labor (n=584); still pregnant at the end of follow-up (n=2933); multiple births (n=686); conception with

Confidential: For Review Only

the help of ART (n=9962); lost to follow-up (n=864); hemoglobin unavailable (n=340). Finally, a total of 34087 women were included in the current analysis. Detailed recruitment criteria of study population were shown in Figure 1.

This study complied with the ethical principles of the Declaration of Helsinki. Ethical approval was given by the Ethics Committee of Xiangya School of Public Health, Central South University (No. XYGW-2018-36). Additionally, it had been registered in Chinese Clinical Trial Registry Center (registration number: ChiCTR1800016635; date of registration: 14 June 2018). Information and biological samples were collected from participants after obtaining written informed consent from all participants.

Information collection

After recruitment, the specially trained investigators used a structured questionnaire to collect the information on maternal sociodemographic characteristics through face-to-face interview. In addition, we gathered data using the database of the Electronic Maternal and Child Health Information System which recorded clinical and biochemical samples from mothers and infants during registration to delivery. The information of maternal complications during pregnancy and pregnancy outcomes of the offspring were obtained from medical records.

Exposures and covariates

The primary exposure was maternal hemoglobin (Hb) concentration during early pregnancy. 5 ml capillary blood sample was collected from pregnant woman in the first trimester (<14 weeks) following standard operating procedures to measure the Hb concentration via a Mindray hematology analyzer available at the Central Laboratory of the Hunan Provincial Maternal and Child Health Care Hospital. Participants whose Hb level during early pregnancy was <11.0 g/dl were considered as anemia according to the WHO definitions.[14] We further categorized the anemia for pregnant women into 3 subgroups (mild anemia: $10.0 \leq Hb < 11.0$ g/dl; moderate anemia: $7.0 \leq Hb < 10.0$ g/dl; severe anemia: $Hb < 7.0$ g/dl).

Information on covariates included socio-demographic characteristics and maternal complications during pregnancy. The time sequence of maternal complications during pregnancy relative to onset of anemia was unknown but might have been associated with outcomes. The covariates for mothers were selected based on literature review as followings: maternal age at pregnancy onset (*< 35 years old and ≥ 35 years*), body mass index (BMI) before pregnancy (*<18.5 kg/m², 18.5-23.9 kg/m², 24-27.9 kg/m² and ≥28 kg/m²*), residence (*urban and rural*), education level (*junior high school or below, senior middle school, college and master or above*), ethnicity (*Han and Minority*), parity (*0, 1-3 and ≥4*), folic acid use before or during pregnancy (*yes or no*), gestational diabetes mellitus (*yes or no*), gestational hypertension (*yes or no*), hyperlipidemia (*yes or no*), hyperthyroidism (*yes or no*), placenta previa (*yes or no*).[15-18]

Outcomes

In this study, the neonatal adverse outcomes of interest were preterm birth, low birth weight, small for gestational age (SGA) and congenital malformation. Preterm birth was defined as delivery before 37 weeks of gestation. Low birth weight was defined as birth weight < 2500g. SGA was defined as birth weight below the 10th percentile of mean weight corrected or fetal sex and gestational age. We categorized congenital malformations based on ICD-10 (International Classification of Diseases 10th revision).

Statistical analysis

Confidential: For Review Only

This study was reported following the STROBE statement. Absolute numbers or percentages was utilized to describe the distributions of maternal characteristics and maternal complications during pregnancy by anemia status. We estimated the rate of each neonatal adverse outcomes in women without anemia and in those with mild, moderate and severe anemia. We did Mantel-Haenszel Chi-square test for trends for each neonatal outcomes to compare trends in the rate of neonatal outcomes by different maternal anemia status. Binary logistic regression model was used to analyze the associations between neonatal outcomes and women with varying status of anemia during early pregnancy. Adjusted odds ratios (aOR) and 95% confidence intervals (95% CIs) were estimated by multivariate logistic regression to control for potential confounders. Sensitivity analyses were conducted by adjusting for different covariates in multivariate models. In model A, we adjusted for maternal age, maternal BMI, residence, education level, ethnicity, parity, and folate use. In model B, we additionally adjusted for complications during pregnancy including gestational diabetes mellitus, gestational hypertension, hyperlipidemia, hyperthyroidism, and placenta previa. EpiData version 3.1 (EpiData Association, Odense, Denmark) was used to develop the database by double entry to ensure the accuracy of the data. All analyses were performed using R software (version 3.5.0). All tests were two-tailed, with P < 0.05 set as the statistically significant difference.

RESULTS

Characteristics of Participants

We included 34,087 pregnant females in our study. A total of 5572 (16.3%) participants were diagnosed with anemia in the first trimester. The prevalence of mild anemia, moderate anemia and severe anemia was 10.9%, 5.2% and 0.3%, respectively. The participants were concentrated in the age group of <35 years (77.5%). 70.2% of the participants had a pre-pregnancy BMI of 18.5-23.9 kg/m^2, and most of individuals lived in urban (61.8%). Their educational level was mostly senior middle school (28.4%) and college (45.7%). Of these individuals, 98.7% were of Han ethnicity, and 1.3% were members of other ethnic groups (includes >50 ethnic groups). A total of 15.9% of the participants were reported a gestational diabetes mellitus, 3.7% had a gestational hypertension, and 1.8% were reported with placenta previa. Distribution of maternal characteristics and complications by anemia status were displayed in Table 1.

Prevalence of neonatal adverse outcomes across maternal anemia status

The prevalence of preterm birth varied by maternal anemia status (Table 2), ranging from 10.9% among women without anemia to 40.0% among women with severe anemia (P_{trend}<0.001). Likewise, the prevalence of low birth weight and SGA infants raised from 7.6% to 44.0% and 14.1% to 35.0% among pregnant females with more severe anemia compared with no anemia, respectively (P_{trend}<0.001). There may be an upward curve relationship between the rate of preterm birth, low birth weight as well as SGA the severity of maternal anemia. Whereas the statistically significant associations between the rate of congenital malformation and different maternal anemia status were not observed (P_{trend}>0.05).

Association of maternal anemia with the risk of neonatal outcomes

To assess the association between the severity of maternal anemia and adverse neonatal outcomes, we compared the risk of the neonatal outcomes for each anemia status to the risk of neonatal outcomes in women without anemia (Hb<11.0 g/dl). After adjusting for potential confounders including age, BMI, residence, education level, ethnicity, parity, and folic acid use (Table 3,

Confidential: For Review Only

model A), the risk estimates of preterm birth (mild anemia aOR 1.61 (95% CI 1.39–1.85), moderate anemia aOR 1.62 (95% CI 1.39–1.88), and severe anemia aOR 1.39 (95% CI 1.17–1.63), respectively), low birth weight (mild anemia aOR 1.61 (95% CI 1.39–1.85), moderate anemia aOR 1.62 (95% CI 1.39–1.88), and severe anemia aOR 1.39 (95% CI 1.17–1.63), respectively) and SGA (mild anemia aOR 1.61 (95% CI 1.39–1.85), moderate anemia aOR 1.62 (95% CI 1.39–1.88), and severe anemia aOR 1.39 (95% CI 1.17–1.63), respectively) significantly increased in offspring of pregnant females who had anemia. However, we did not find that women with anemia were associated with the risk of congenital malformation in offspring. Adjusting for different covariates did not substantially affect the risk estimates of adverse neonatal outcomes, which were similar for model A and model B.

DISCUSSION

We conducted a prospective cohort study using information about pregnant women in Hunan provincial Maternal and Child Health Care Hospital, China. The prevalence of gestational anemia was 16.3% in the present study which was comparable with results previously reported in China.[19, 20] After adjusting for maternal confounders, anemia during early pregnancy was associated with the risk of preterm birth, low birth weight and SGA. Association between the risk of congenital malformation and anemia during early pregnancy was not observed. Sensitivity analysis verified the stability of the results. Furthermore, an upward curve for the rate of preterm birth, low birth weight and SGA with the severity of maternal anemia in the first trimester was identified.

Published literature had already reported associations of anemia during pregnancy with neonatal outcomes such as preterm birth, low birth weight and SGA. However, these findings had not been consistent.[21-23] This might be that various researches defined anemia during pregnancy with different cutoff thresholds. Besides, the associations between maternal anemia and neonatal outcomes varied depending on the timing of the maternal Hb measurement.[24] In the current study, we specified that Hb was measured in the first trimester to analyze the associations between anemia during early pregnancy and neonatal outcomes. Our data showed that maternal anemia during early pregnancy had negative effects on preterm birth, low birth weight and SGA. Consistent with our findings, a meta-analysis reported an increased risk of low birth weight among women with lower Hb concentrations during overall or any stage of pregnancy.[8] Associations between maternal anemia in the first trimester and neonatal outcomes was inconclusive. According to the results of Ronkainen *et al.*,[25] the risk of SGA increased in offspring of pregnant females whose level of Hb was <11.0 g/dl in the first trimester. But this study showed that no association between low maternal Hb level and preterm birth. A study performed in the UK came to the same conclusion that low maternal Hb during early pregnancy associated with SGA, but not preterm birth.[26] However, Ren's result implied that rates of low birth weight, preterm birth, and SGA increased with the decrease in first-trimester Hb concentration in this Chinese population which was consistent with our research results.[27]

Hb carries oxygen to the placenta, which is essential for normal fetal growth in the uterus. Anemia or low Hb levels may stimulate changes in placental angiogenesis and affect placental transit function.[28] As a result, it reduces the oxygen supply and nutrients supplementation to the fetus, resulting in intrauterine growth retardation and low birth weight.[29] Increased placental vascularization in women with low Hb levels suggests earlier placental maturation, which may

Confidential: For Review Only

contribute to an increased risk of preterm birth.[30] In addition, low Hb may lead to chronic hypoxia which in turn may cause a stress response of increased placental corticotropin-releasing hormone secretion, inducing adverse consequences such as preterm birth, gestational hypertension and so forth.[31] Nevertheless, the underlying biological mechanisms involved in the associations between maternal anemia and preterm birth, low birth weight as well as SGA remain to be further elucidated.

Organogenesis occurs in the first trimester of pregnancy, during which the primary structures of most organs develop such as the heart, lung, liver, and so on. In the early stage of development, the embryo and the fetus undergo intense epigenetic reprogramming.[32, 33] Previous studies have shown that epigenetic changes might play a significant role in offspring diseases caused by gestational complications.[34] Gestational anemia is one of the most common gestational complications. Our study revealed lack of association between maternal anemia during early pregnancy and congenital malformation. Shi *et al.*[35] also found that no association between low birth weight and maternal anemia during overall pregnancy.

This study utilized a large sample of pregnant women and took place a prospective cohort study which minimized recall bias and ensured the reliability of the study. Additionally, our study specified that Hb was measured during the early pregnancy to analyze the association between anemia during early pregnancy and neonatal outcomes. However, some limitations should be considered. Firstly, the study objects were all from the same hospital, and the sample sources may be concentrated in a certain group of population. This may affect the representativeness of the sample and thus affect extrapolation. Secondly, although adjusting for a few potential confounding factors, we did not measure several important factors, especially iron supplementation and other nutritional conditions during pregnancy. Thirdly, we could not determine the cause of maternal anemia so we could not distinguish which specific type of anemia was associated with neonatal outcomes.

CONCLUSION

In conclusion, it is of concern that anemia during the early pregnancy regardless of the severity may significantly increase the risk of neonatal outcomes such as preterm birth, low birth weight and SGA. According to the results of our study, we suggest that the rate of neonatal outcomes may increase with the severity of maternal anemia. However, more prospective studies with larger samples are needed to verify the authenticity of the results and explore the underlying mechanisms. Pre-pregnancy screening for anemia and implementation of interventions are necessary to ensure adequate Hb concentrations during pregnancy and to reduce the adverse effects of maternal anemia on the health of their offspring.

Funding

This work was supported by the Project Funded by National Natural Science Foundation Program of China (82073653 and 81803313), China Postdoctoral Science Foundation (2020M682644), Hunan Provincial Science and Technology Talent Support Project (2020TJ-N07), Hunan Provincial Key Research and Development Program (2018SK2063), Open Project from NHC Key Laboratory of Birth Defect for Research and Prevention (KF2020006), Natural Science

Confidential: For Review Only

Foundation of Hunan Province (2018JJ2551) and Science and Technology Planning Project of Guangdong Province (2020A1414010152).

Author Contributions

YPL and TWZ wrote the main manuscript text; YPL and TTW analyzed the data statistical analyses; JBQ and TBY reviewed and revised the manuscript; XYL, YPL, XLS, TTW, SMZ, MTS, JHW and JS have collected the data. All authors have read and approved the final version of the manuscript.

Patient and Public Involvement Statement

Patients or the public were involved in the design, or conduct, or reporting, or dissemination plans of our research. Refer to the Methods section for further details.

Patient consent for publication

Not required.

Ethics approval

This study was approved by the ethics committee of the Xiangya School of Public Health of Central South University, and registered in the Chinese Clinical Trial Registry (Registration number: ChiCTR1800016635).

Provenance and peer review

Not commissioned; externally peer reviewed.

Data Availability Statement

The datasets used and/or analyzed during the current study are available from the corresponding author on reasonable request.

Acknowledgments

The authors would like to thank the editors and reviewers for their suggestions and all colleagues working in Maternal and Child Health Promotion and Birth Defect Prevention Group.

Conflict of Interest

The authors declared no potential conflicts of interest with respect to the research, authorship, and/or publication of this article.

Reference

1. Balarajan, Y.; Ramakrishnan, U.; Ozaltin, E.; Shankar, A.H.; Subramanian, S.V. Anaemia in low-income and middle-income countries. *Lancet*. **2011**, *378*, 2123-2135.
2. McLean, E.; Cogswell, M.; Egli, I.; Wojdyla, D.; de Benoist, B. Worldwide prevalence of anaemia, WHO Vitamin and Mineral Nutrition Information System, 1993-2005. *Public Health Nutr*. **2009**, *12*, 444-454.
3. Black, R.E.; Victora, C.G.; Walker, S.P.; Bhutta, Z.A.; Christian, P.; de Onis, M.; Ezzati, M.; Grantham-McGregor, S.; Katz, J.; Martorell, R.; Uauy, R. Maternal and child undernutrition and overweight in low-income and middle-income countries. *Lancet*. **2013**, *382*, 427-451.
4. Stevens, G.A.; Finucane, M.M.; De-Regil, L.M.; Paciorek, C.J.; Flaxman, S.R.; Branca, F.; Peña-Rosas, J.P.; Bhutta, Z.A.; Ezzati, M. Global, regional, and national trends in haemoglobin concentration and prevalence of total and severe anaemia in children and pregnant and non-pregnant women for 1995-2011: a systematic analysis of population-representative data. *Lancet Glob Health*. **2013**, *1*, e16-e25.
5. Organization, N.; Health, W. Global nutrition targets 2025: Anaemia policy brief. *Working*

Confidential: For Review Only

Papers. **2016**.

6. Haider, B.A.; Olofin, I.; Wang, M.; Spiegelman, D.; Ezzati, M.; Fawzi, W.W. Anaemia, prenatal iron use, and risk of adverse pregnancy outcomes: systematic review and meta-analysis. *BMJ*. **2013**, *346*, f3443.
7. Jung, J.; Rahman, M.M.; Rahman, M.S.; Swe, K.T.; Islam, M.R.; Rahman, M.O.; Akter, S. Effects of hemoglobin levels during pregnancy on adverse maternal and infant outcomes: a systematic review and meta-analysis. *Ann N Y Acad Sci*. **2019**, *1450*, 69-82.
8. Young, M.F.; Oaks, B.M.; Tandon, S.; Martorell, R.; Dewey, K.G.; Wendt, A.S. Maternal hemoglobin concentrations across pregnancy and maternal and child health: a systematic review and meta-analysis. *Ann N Y Acad Sci*. **2019**, *1450*, 47-68.
9. Malhotra, M.; Sharma, J.B.; Batra, S.; Sharma, S.; Murthy, N.S.; Arora, R. Maternal and perinatal outcome in varying degrees of anemia. *Int J Gynaecol Obstet*. **2002**, *79*, 93-100.
10. Bora, R.; Sable, C.; Wolfson, J.; Boro, K.; Rao, R. Prevalence of anemia in pregnant women and its effect on neonatal outcomes in Northeast India. *J Matern Fetal Neonatal Med*. **2014**, *27*, 887-891.
11. Menon, K.C.; Ferguson, E.L.; Thomson, C.D.; Gray, A.R.; Zodpey, S.; Saraf, A.; Das, P.K.; Skeaff, S.A. Effects of anemia at different stages of gestation on infant outcomes. *Nutrition*. **2016**, *32*, 61-65.
12. de Haas, S.; Ghossein-Doha, C.; van Kuijk, S.M.; van Drongelen, J.; Spaanderman, M.E. Physiological adaptation of maternal plasma volume during pregnancy: a systematic review and meta-analysis. *Ultrasound Obstet Gynecol*. **2017**, *49*, 177-187.
13. Díaz-López, A.; Ribot, B.; Basora, J.; Arija, V. High and Low Haemoglobin Levels in Early Pregnancy Are Associated to a Higher Risk of Miscarriage: A Population-Based Cohort Study. *Nutrients*. **2021**, *13*.
14. Organization, W.H. EN VMNIS - Indicators - Haemoglobin concentrations for the diagnosis of anaemia and assessment of severity. **2011**.
15. Ananth, C.V.; VanderWeele, T.J. Placental abruption and perinatal mortality with preterm delivery as a mediator: disentangling direct and indirect effects. *Am. J. Epidemiol.* **2011**, *174*, 99-108.
16. Fadl, N.; Ice, G.H.; Haile, Z.T. Association between maternal high-risk factors and anemia among women in Ethiopia. *Nutrition*. **2021**, *91-92*, 111404.
17. Sharma, D.; Amgain, K.; Panta, P.P.; Pokhrel, B. Hemoglobin levels and anemia evaluation among pregnant women in the remote and rural high lands of mid-western Nepal: a hospital based study. *BMC Pregnancy Childbirth*. **2020**, *20*, 182.
18. Churchill, D.; Nair, M.; Stanworth, S.J.; Knight, M. The change in haemoglobin concentration between the first and third trimesters of pregnancy: a population study. *BMC Pregnancy Childbirth*. **2019**, *19*, 359.
19. Tan, J.; He, G.; Qi, Y.; Yang, H.; Xiong, Y.; Liu, C.; Wang, W.; Zou, K.; Lee, A.H.; Sun, X.; Liu, X. Prevalence of anemia and iron deficiency anemia in Chinese pregnant women (IRON WOMEN): a national cross-sectional survey. *BMC Pregnancy Childbirth*. **2020**, *20*, 670.
20. Wu, Y.; Ye, H.; Liu, J.; Ma, Q.; Yuan, Y.; Pang, Q.; Liu, J.; Kong, C.; Liu, M. Prevalence of anemia and sociodemographic characteristics among pregnant and non-pregnant women in southwest China: a longitudinal observational study. *BMC Pregnancy Childbirth*. **2020**, *20*, 535.

Confidential: For Review Only

21. Räisänen, S.; Kancherla, V.; Gissler, M.; Kramer, M.R.; Heinonen, S. Adverse perinatal outcomes associated with moderate or severe maternal anaemia based on parity in Finland during 2006-10. *Paediatr Perinat Epidemiol*. **2014**, *28*, 372-380.
22. Ali, S.A.; Tikmani, S.S.; Saleem, S.; Patel, A.B.; Hibberd, P.L.; Goudar, S.S.; Dhaded, S.; Derman, R.J.; Moore, J.L.; McClure, E.M.; Goldenberg, R.L. Hemoglobin concentrations and adverse birth outcomes in South Asian pregnant women: findings from a prospective Maternal and Neonatal Health Registry. *Reprod. Health*. **2020**, *17*, 154.
23. Zhang, X.; Xu, Q.; Yang, Y.; Wang, L.; Liu, F.; Li, Q.; Ji, M.; He, Y.; Wang, Y.; Zhang, Y.; Zhang, H.; Peng, Z.; Ma, X.; Yu, Z. Preconception Hb concentration and risk of preterm birth in over 2·7 million Chinese women aged 20-49 years: a population-based cohort study. *Br J Nutr*. **2018**, *120*, 508-516.
24. Liu, D.; Li, S.; Zhang, B.; Kang, Y.; Cheng, Y.; Zeng, L.; Chen, F.; Mi, B.; Qu, P.; Zhao, D.; Zhu, Z.; Yan, H.; Wang, D.; Dang, S. Maternal Hemoglobin Concentrations and Birth Weight, Low Birth Weight (LBW), and Small for Gestational Age (SGA): Findings from a Prospective Study in Northwest China. *Nutrients*. **2022**, *14*.
25. Ronkainen, J.; Lowry, E.; Heiskala, A.; Uusitalo, I.; Koivunen, P.; Kajantie, E.; Vääräsmäki, M.; Järvelin, M.R.; Sebert, S. Maternal hemoglobin associates with preterm delivery and small for gestational age in two Finnish birth cohorts. *Eur J Obstet Gynecol Reprod Biol*. **2019**, *238*, 44-48.
26. Alwan, N.A.; Cade, J.E.; McArdle, H.J.; Greenwood, D.C.; Hayes, H.E.; Simpson, N.A. Maternal iron status in early pregnancy and birth outcomes: insights from the Baby's Vascular health and Iron in Pregnancy study. *Br J Nutr*. **2015**, *113*, 1985-1992.
27. Ren, A.; Wang, J.; Ye, R.W.; Li, S.; Liu, J.M.; Li, Z. Low first-trimester hemoglobin and low birth weight, preterm birth and small for gestational age newborns. *Int J Gynaecol Obstet*. **2007**, *98*, 124-128.
28. Figueiredo, A.; Gomes-Filho, I.S.; Batista, J.; Orrico, G.S.; Porto, E.; Cruz, P.R.; Dos, S.C.S.; Brito, S.M.; Ramos, M.; Sena, M.; Vilasboas, S.; Seixas, D.C.S.; Pereira, M.G. Maternal anemia and birth weight: A prospective cohort study. *Plos One*. **2019**, *14*, e212817.
29. Chu F. C., Shen-Wen Shao S., Lo L.-M., Hsieh T., Hung T. H. Association between maternal anemia at admission for delivery and adverse perinatal outcomes. *Journal of Chinese Medical Association*. **2020**, 83, 402–407.
29. Stangret, A.; Wnuk, A.; Szewczyk, G.; Pyzlak, M.; Szukiewicz, D. Maternal hemoglobin concentration and hematocrit values may affect fetus development by influencing placental angiogenesis. *J Matern Fetal Neonatal Med*. **2017**, *30*, 199-204.
30. Bakacak, M.; Avci, F.; Ercan, O.; Köstü, B.; Serin, S.; Kiran, G.; Bostanci, M.S.; Bakacak, Z. The effect of maternal hemoglobin concentration on fetal birth weight according to trimesters. *J Matern Fetal Neonatal Med*. **2015**, *28*, 2106-2110.
31. Liu, X.; Wang, C.; Liu, W.; Li, J.; Li, C.; Kou, X.; Chen, J.; Zhao, Y.; Gao, H.; Wang, H.; Zhang, Y.; Gao, Y.; Gao, S. Distinct features of H3K4me3 and H3K27me3 chromatin domains in pre-implantation embryos. *Nature*. **2016**, *537*, 558-562.
32. Wang, C.; Liu, X.; Gao, Y.; Yang, L.; Li, C.; Liu, W.; Chen, C.; Kou, X.; Zhao, Y.; Chen, J.; Wang, Y.; Le R; Wang, H.; Duan, T.; Zhang, Y.; Gao, S. Reprogramming of H3K9me3-dependent heterochromatin during mammalian embryo development. *Nat. Cell Biol*. **2018**, *20*, 620-631.

Confidential: For Review Only

33. Sun, C.F.; Liu, H.; Hao, Y.H.; Hu, H.T.; Zhou, Z.Y.; Zou, K.X.; Liu, X.M.; Sheng, J.Z.; Ding, G.L.; Huang, H.F. Association between gestational anemia in different trimesters and neonatal outcomes: a retrospective longitudinal cohort study. *World J. Pediatr.* **2021**, *17*, 197-204.

34. Shi, H.; Chen, L.; Wang, Y.; Sun, M.; Guo, Y.; Ma, S.; Wang, X.; Jiang, H.; Wang, X.; Lu, J.; Ge, L.; Dong, S.; Zhuang, Y.; Zhao, Y.; Wei, Y.; Ma, X.; Qiao, J. Severity of Anemia During Pregnancy and Adverse Maternal and Fetal Outcomes. *JAMA Netw Open*. **2022**, *5*, e2147046.

Figure legend:

Figure 1: Flow chart showing the process of participant's recruitment

Confidential: For Review Only

1 Table 1 Distribution of maternal characteristics and complications by anemia status

Characteristics	Total N (%)	Maternal hemoglobin, N (%)			
		Normal ≥11.0 g/dl	Mild 10.0-10.9 g/dl	Moderate 7.0–9.9 g/dl	Severe <7.0 g/dl
Deliveries	34087	28515 (83.7)	3703 (10.9)	1769 (5.2)	100 (0.3)
Maternal age (years)					
<35	26433 (77.5)	22082 (77.4)	2890 (78.0)	1382 (78.1)	79 (79.0)
≥35	7654 (22.5)	6433 (22.6)	813 (22.0)	387 (21.9)	21 (21.0)
Maternal BMI					
<18.5	4920 (14.4)	3978 (14.0)	574 (15.5)	336 (19.0)	32 (32.0)
18.5-23.9	23913 (70.2)	20123 (70.6)	2547 (68.8)	1184(76.9)	59 (59.0)
24-27.9	4329(12.7)	3653 (12.8)	472 (12.7)	195 (11.0)	9 (9.0)
≥28	925 (2.7)	761 (2.7)	110 (3.0)	54 (3.1)	0 (0.0)
Residence					
Urban	21062 (61.8)	17672 (62.0)	2253 (60.8)	1082 (61.2)	55 (55.0)
Rural	13025 (38.2)	10843 (38.0)	1450 (39.2)	687 (38.8)	45 (45.0)
Education level					
Junior high school or below	2547 (7.5)	1705 (6.0)	516 (13.9)	299 (16.9)	27 (27.0)
Senior middle school	9684 (28.4)	7842 (27.5)	1218 (32.9)	594 (33.6)	30 (30.0)
College	15588 (45.7)	13618 (47.8)	1346 (36.3)	586 (33.1)	38 (38.0)
Master or above	6268 (18.4)	5350 (18.8)	623 (18.8)	290 (16.4)	5 (5.0)
Ethnicity					
Han	33639 (98.7)	28129 (98.6)	3686 (99.5)	1730 (97.8)	94 (94.0)
Minority	448 (1.3)	386 (1.4)	17 (0.5)	39 (2.2)	6 (6.0)
Parity					
0	10488 (30.8)	9274 (32.5)	813 (22.0)	369 (20.9)	32 (32.0)
1-3	20189 (59.2)	16601 (58.2)	2362 (62.8)	1168 (66.0)	58 (58.0)
≥4	3410 (10.0)	2640 (9.3)	528 (14.3)	232 (13.1)	10 (10.0)
Folic acid use *	32540 (95.5)	27272 (95.6)	3490 (94.2)	1687 (95.4)	91 (91.0)
Gestational diabetes mellitus	5424 (15.9)	4689 (16.4)	485 (13.1)	250 (14.1)	0 (0.0)
Gestational hypertension	1246 (3.7)	1012 (3.5)	161 (4.3)	68 (3.8)	5 (5.0)
Hyperlipidemia	375 (1.1)	312 (1.1)	35 (0.9)	28 (1.6)	0 (0.0)
Hyperthyroidism	636 (1.9)	542 (1.9)	71 (1.9)	23 (1.3)	0 (0.0)
Placenta previa	606 (1.8)	492 (1.7)	70 (1.9)	42 (2.4)	2 (2.0)

2 BMI = body mass index
3 * Folic acid use before or during pregnancy

4

5

Confidential: For Review Only

1Table 2 Prevalence (% and 95% CI) of neonatal adverse outcomes according to maternal anemia status

Maternal anemia	Preterm birth % (95% CI)	Low birthweight % (95% CI)	SGA % (95% CI)	Congenital malformation % (95% CI)
Normal (≥11.0 g/dl)	10.9 (10.5-11.2)	7.6 (7.2-7.9)	14.1 (13.7-14.5)	4.4 (4.2-4.7)
Mild (10.0-10.9 g/dl)	15.7 (14.5-16.9)	13.7 (12.6-14.8)	20.2 (18.9-21.5)	3.9 (3.3-4.6)
Moderate (7.0–9.9 g/dl)	18.1 (16.3-19.9)	17.8 (16.0-19.6)	22.8 (20.8-24.7)	5.0 (4.0-6.0)
Severe (<7.0 g/dl)	40.0 (30.2-49.8)	44.0 (34.1-53.9)	35.0 (25.5-44.5)	4.0 (0.1-7.9)
P_{trend}	<0.001	<0.001	<0.001	0.899

2 SGA= small for gestational age; 95% CI = 95% confidence interval.

3

4

Confidential: For Review Only

Table 3 Adjusted risk ratios for neonatal adverse outcomes associated with severity of anemia during early pregnancy [a]

Outcome	Model A[b]				Model B[c]			
	Mild 10.0-10.9 g/dl	Moderate 7.0–9.9 g/dl	Severe <7.0 g/dl	Overall <11.0 g/dl	Mild 10.0-10.9 g/dl	Moderate 7.0–9.9 g/dl	Severe <7.0 g/dl	Overall <11.0 g/dl
Preterm birth	1.43(1.30-1.57) *	1.67(1.47-1.90) *	4.66(3.10-7.01) *	1.55(1.43-1.68) *	1.42(1.29-1.56) *	1.67(1.47-1.90) *	4.66(3.09-7.02) *	1.54(1.42-1.67) *
Low birthweight	1.71(1.53-1.90) *	2.31(2.03-2.65) *	7.63(5.03-11.58) *	1.97(1.80-2.15) *	1.70(1.52-1.89) *	2.33(2.04-2.67) *	7.82(5.12-11.94) *	1.97(1.80-2.12) *
SGA	1.41(1.30-1.54) *	1.62(1.44-1.82) *	2.76(1.82-4.19) *	1.50(1.39-1.61) *	1.42(1.30-1.55) *	1.62(1.44-1.82) *	2.81(1.85-4.27) *	1.50(1.40-1.62) *
Congenital malformation	0.89(0.75-1.06)	1.14(0.91-1.42)	0.83(0.30-2.26)	0.97(0.84-1.12)	0.88(0.74-1.05)	1.12(0.89-1.40)	0.80(0.29-2.19)	0.96(0.83-1.10)

SGA= small for gestational age.

[a] Non-anemic pregnant women as reference.

[b] Model A adjusted for maternal age, maternal BMI, residence, education level, ethnicity, parity, and folate use.

[c] Model B adjusted for all maternal complications during pregnancy in addition to covariates in model A.

* $P < 0.005$.

Confidential: For Review Only

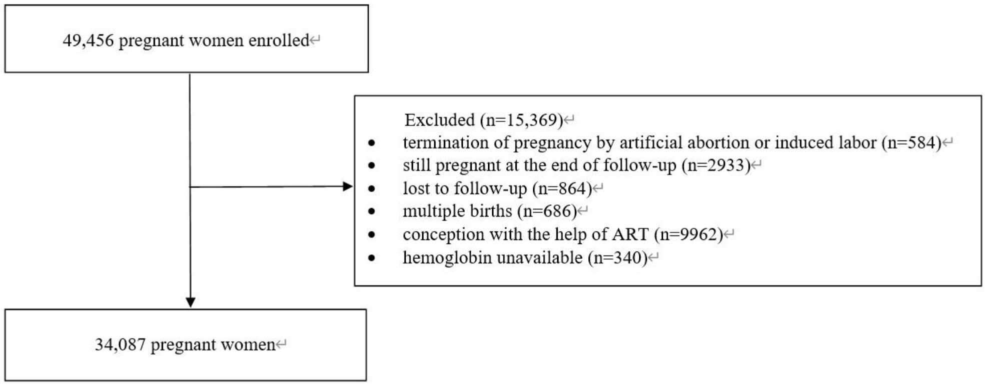

Flow chart showing the process of participant's recruitment

187x80mm (168 x 168 DPI)

BMJ Paediatrics Open

Maternal anemia during early pregnancy and the risk of neonatal outcomes: a prospective cohort study in Central China

Journal:	*BMJ Paediatrics Open*
Manuscript ID	bmjpo-2023-001931.R1
Article Type:	Original research
Date Submitted by the Author:	09-May-2023
Complete List of Authors:	Chen, Yige; Central South University, Department of Epidemiology and Health Statistics, Xiangya School of Public Health<br>Zhong, Taowei; Central South University, Department of Epidemiology and Health Statistics<br>Song, Xinli; Central South University, Department of Epidemiology and Health Statistics<br>Zhang, Senmao; Central South University, Department of Epidemiology and Health Statistics<br>Sun, Mengting; Central South University, Department of Epidemiology and Health Statistics<br>Liu, Xiaoying; Changsha Medical University, Public Health Institute; The Hospital of Trade-Business in Hunan Province<br>Wei, Jianhui; Central South University, Department of Epidemiology and Health Statistics<br>Shu, Jing; Central South University, Department of Epidemiology and Health Statistics<br>Liu, Yiping; Central South University, Department of Epidemiology and Health Statistics<br>Qin, Jiabi; Central South University, Department of Epidemiology and Health Statistics; Hunan Provincial Maternal and Child Health Care Hospital
Keywords:	Epidemiology, Growth, Neonatology

SCHOLARONE™
Manuscripts

I, the Submitting Author has the right to grant and does grant on behalf of all authors of the Work (as defined in the below author licence), an exclusive licence and/or a non-exclusive licence for contributions from authors who are: i) UK Crown employees; ii) where BMJ has agreed a CC-BY licence shall apply, and/or iii) in accordance with the terms applicable for US Federal Government officers or employees acting as part of their official duties; on a worldwide, perpetual, irrevocable, royalty-free basis to BMJ Publishing Group Ltd ("BMJ") its licensees and where the relevant Journal is co-owned by BMJ to the co-owners of the Journal, to publish the Work in this journal and any other BMJ products and to exploit all rights, as set out in our licence.

The Submitting Author accepts and understands that any supply made under these terms is made by BMJ to the Submitting Author unless you are acting as an employee on behalf of your employer or a postgraduate student of an affiliated institution which is paying any applicable article publishing charge ("APC") for Open Access articles. Where the Submitting Author wishes to make the Work available on an Open Access basis (and intends to pay the relevant APC), the terms of reuse of such Open Access shall be governed by a Creative Commons licence – details of these licences and which Creative Commons licence will apply to this Work are set out in our licence referred to above.

Other than as permitted in any relevant BMJ Author's Self Archiving Policies, I confirm this Work has not been accepted for publication elsewhere, is not being considered for publication elsewhere and does not duplicate material already published. I confirm all authors consent to publication of this Work and authorise the granting of this licence.

Confidential: For Review Only

Maternal anemia during early pregnancy and the risk of neonatal outcomes: a prospective cohort study in Central China

Yige Chen[1, 2†], Taowei Zhong[1†], Xinli Song[1], Senmao Zhang[1], Mengting Sun[1], Xiaoying Liu[3, 4], Jianhui Wei[1], Jing Shu[1], Yiping Liu[1*], Jiabi Qin[1, 5*]

[1] Department of Epidemiology and Health Statistics, Xiangya School of Public Health, Central South University, Changsha, Hunan, China

[2] epartment of Science and Education, Xiangya Changde, Hospital, Changde, China

[3] Public Health Institute, Changsha Medical University, Changsha, Hunan, China

[4] The Hospital of Trade-Business in Hunan Province, Changsha, Hunan, China

[5] Hunan Provincial Key Laboratory of Clinical Epidemiology, Changsha, Hunan, China

[†] **Co-First Author:** Yige Chen, Taowei Zhong

[*] **Co-Corresponding Author**

Yiping Liu, Department of Epidemiology and Health Statistics, Xiangya School of Public Health, Central South University, 110 Xiangya Road, Changsha, Hunan 410078, China;

Telephone: (+86)18843113354; Email: 18843113354@163.com;

Jiabi Qin, Department of Epidemiology and Health Statistics, Xiangya School of Public Health, Central South University, 110 Xiangya Road, Changsha, Hunan 410078, China;

Telephone: (+86)15974269544; E-mail: qinjiabi123@163.com.

Word count:3187 words

Confidential: For Review Only

Abstract

Background: The purpose of this study was to explore the association between anemia during early pregnancy and the risk of neonatal outcomes.

Methods: We collected clinical data from pregnant females (≥18 years) who received their first antenatal care between 8-14 weeks of gestation in Hunan Provincial Maternal and Child Health Care Hospital. Multiple logistic regression models and restricted cubic spline regression models were used to analyze the association between anemia during early pregnancy and the risk of neonatal outcomes. In addition, sensitivity analysis was further performed to assess the robustness of the results.

Results: The prospective cohort study ultimately included 34,087 singleton pregnancies. In this study, the rate of anemia during early pregnancy was 16.3%. Our data showed that there was a positive relationship between the rate of preterm birth, low birth weight as well as SGA and the severity of maternal anemia (P_{trend}<0.05). After adjustment, the association of early pregnancy anemia and Hb levels with the the risk of preterm birth (mild anemia aOR 1.37 (95% CI 1.25-1.52), moderate anemia aOR 1.54 (95% CI 1.35-1.76), and severe anemia aOR 4.03 (95% CI 2.67-6.08), respectively), low birth weight (mild anemia aOR 1.61 (95% CI 1.44-1.79), moderate anemia aOR 2.01 (95% CI 1.75-2.30), and severe anemia aOR 6.11 (95% CI 3.99-9.36), respectively) and SGA (mild anemia aOR 1.37 (95% CI 1.25-1.52), moderate anemia aOR 1.54 (95% CI 1.35-1.76), and severe anemia aOR 2.61 (95% CI 1.74-4.50), respectively; $P_{non-linear}$<0.05) was observed. However, no association was found between early pregnancy anemia or Hb levels and the risk of congenital malformations. Sensitivity analysis verified the stability of the results.

Conclusions: Maternal anemia during early pregnancy was associated with an increased risk of preterm birth, low birth weight and SGA, and their rates may increase with the severity of maternal anemia.

Trial registration: This study was registered in the Chinese Clinical Trial Registry (Registration number: ChiCTR1800016635).

Keywords: Epidemiology, Growth, Neonatology

Confidential: For Review Only

What is known about the subject

- Anemia is the most common nutritional deficiency in pregnancy across the world.
- Maternal anemia is related to adverse neonatal outcomes, but there are inconsistent findings.
- These associations of maternal anemia with adverse neonatal outcomes may vary when the severity of gestational anemia is considered.

What this study adds:

- Maternal anemia in the first trimester (8-14 weeks) is associated with an increased risk of preterm birth, low birth weight and small for gestational age (SGA).
- There is a positive relationship between the rate of preterm birth, low birth weight as well as SGA and the severity of maternal anemia.
- Non-linear associations of maternal Hb concentrations with risk of preterm birth, low birth weight and SGA in offspring are observed.

How this study may affect research, practice or policy

- This study may ensure the reliability of the relationship between maternal anemia and adverse neonatal outcomes.
- To involve pregnant women with different severity of anemia in timely prenatal care may be beneficial to reducing the risk of adverse neonatal outcomes.

Confidential: For Review Only

INTRODUCTION

Anemia is a pathophysiological condition in which the level of hemoglobin (Hb) and/or the quantity of red blood cells are lower than established threshold values, thereby impairing the capacity of the oxygen-carrying to tissues[1]. It has been claimed to be the most common nutritional deficiency in pregnancy across the world, bringing a high burden to developed and developing countries[2, 3]. Anemia in pregnancy constitutes a global public health challenge with its high prevalence. Stevens *et al.*[4] reported that the global prevalence of anemia in pregnant women was 38%, affecting approximately 32 million individuals. The significance of reducing and properly managing maternal anemia has been recognized globally. Current targets which set in Sustainable Development Goal 3 and Global Nutrition Targets 2025 call for a 50% decrease of anemia in women of reproductive age by the year 2025[5].

Anemia during pregnancy is a key contributor to adverse neonatal outcomes[6]. Findings have widely demonstrated associations between maternal anemia and adverse neonatal outcomes, such as low birth weight, preterm birth, and small for gestational age (SGA)[7, 8]. But these associations may be different when the severity of gestational anemia is taken into account. Limited data suggested that risks of low birth weight decreased in the offspring of pregnant women who have mild anemia compared with pregnant women who have no anemia[9]. A study of 421 pregnant women in India indicated that an association between the severity of anemia and low birth weight as well as SGA[10].

The Hb concentration of pregnant women decreases from the second trimester[11]. This is due to the reason that the expansion of plasma volume begins in the second trimester of pregnancy physiologically[12]. Therefore, measurements of Hb made during or after the second trimester are likely to overvalue the prevalence of anemia. The level of Hb estimated before the 14th week of gestation may reflect Hb levels in pre-pregnancy women and guide future timing and delivery of maternal interventions and prenatal care[13]. In addition, organogenesis occurs in first-trimester pregnancy, during which embryos and fetuses are liable to intense epigenetic reprogramming. It has been suggested that gestational complications, intrauterine environments and a maternal inadequate nutritional status could ultimately result in a range of adverse neonatal outcomes via epigenetic change[14-16]. What's more, anemia during the first trimester was more detrimental[17]. Overall, anemia in the first trimester of pregnancy is more likely to lead to adverse neonatal outcomes and is more harmful than anemia in other trimesters. So, we took maternal anemia in the first trimester for analysis in this study.

To sum up, this study was based on a prospective cohort study to further clarify the association between the severity of anemia during early pregnancy and the risk of adverse neonatal outcomes in China.

METHODS

Recruitment of study participants

A prospective cohort study design was implemented. From March 13, 2013 to January 31, 2020, pregnant females (≥18 years) who received their first antenatal care at 8-14 weeks of gestation with the intention of continuously receiving prenatal care throughout gestation at Hunan Provincial Maternal and Child Health Care Hospital, in Hunan Province, China were approached and recruited into this cohort. Gestational weeks were based on the last menstrual period data, or estimated by ultrasonography if menstruation was irregular. During these pregnant women's first

Confidential: For Review Only

antenatal visit, a total of 49,456 pregnant females met these inclusion criteria were invited to involve in this present cohort. Considering assisted reproductive technology (ART) may lead to misestimation of the prevalence of preterm birth and low birth weight, women concepted with the help of ART were not included in our study. We excluded 15,369 women on the strength of the following exclusion criteria: termination of pregnancy by artificial abortion or induced labor (n=584); still pregnant at the end of follow-up (n=2,933); multiple births (n=686); conception with the help of ART (n=9,962); lost to follow-up (n=864); hemoglobin unavailable (n=340). Finally, a total of 34,087 women were included in the current analysis. Detailed recruitment criteria of study population were shown in Figure 1.

This study complied with the ethical principles of the Declaration of Helsinki. Ethical approval was given by the Ethics Committee of Xiangya School of Public Health, Central South University (No. XYGW-2018-36). Additionally, it had been registered in Chinese Clinical Trial Registry Center (registration number: ChiCTR1800016635; date of registration: 14 June 2018). Information and biological samples were collected from participants after obtaining written informed consent from all participants.

Information collection

After recruitment, the specially trained investigators used a structured questionnaire to collect information on maternal sociodemographic characteristics through face-to-face interviews. In addition, we gathered data using the database of the Electronic Maternal and Child Health Information System which recorded clinical and biochemical samples from mothers and infants during registration to delivery. The information on maternal complications during pregnancy and pregnancy outcomes of the offspring were obtained from medical records.

Exposures and covariates

The primary exposure was maternal hemoglobin (Hb) concentration in 1st trimester (<14 weeks). Pregnant women accepted their first antenatal care at 8-14 gestational weeks and were concurrently collected 5 ml blood samples following standard operating procedures to measure the Hb concentration via a Mindray hematology analyzer available at the Central Laboratory of the Hunan Provincial Maternal and Child Health Care Hospital. Participants whose Hb level during early pregnancy was <110 g/L were considered as anemia according to the WHO definitions[18]. We further categorized the anemia for pregnant women into 3 subgroups (mild anemia: 10.0≤ Hb<110 g/L; moderate anemia: 70≤ Hb <100 g/L; severe anemia: Hb <70 g/L).

Information on covariates included socio-demographic characteristics and maternal complications during pregnancy. The covariates for mothers were selected based on literature review as followings: maternal age at pregnancy onset (*< 35 years old and ≥ 35 years*), body mass index (BMI) before pregnancy (*<18.5 kg/m², 18.5-23.9 kg/m², 24-27.9 kg/m² and ≥28 kg/m²*), residence (*urban and rural*), education level (*junior high school or below, senior middle school, college and master or above*), ethnicity (*Han and Minority*), parity (*0, 1-3 and ≥4*), folic acid use before or during pregnancy (*yes or no,*), per caput monthly family income (*≤2500, 2500-5000, >5000, RMB*), gestational diabetes mellitus (*yes or no*), gestational hypertension (*yes or no*), hyperlipidemia (*yes or no*), hyperthyroidism (*yes or no*), placenta previa (*yes or no*)[19-22]. Folic acid use before or during pregnancy is defined as taking at least 0.4 mg of folic acid daily for more than five days per week continuously before conception or throughout the first trimester of pregnancy[23]. In China, a Maternal and Child Health Manual will be offered to every pregnant woman and recorded the following information, including the basic demographic characteristics,

Confidential: For Review Only

clinical examination, and folic acid supplementation. After finishing the questionnaire, the information in the manual will be compared to the questionnaire to confirm the accuracy of gathered information.

Outcomes

In this study, the neonatal adverse outcomes of interest were preterm birth, low birth weight, small for gestational age (SGA) and congenital malformation. Preterm birth was defined as delivery before 37 weeks of gestation. Low birth weight was defined as birth weight < 2500g. SGA was defined as birth weight below the 10th percentile of mean weight corrected or fetal sex and gestational age. We categorized congenital malformations based on ICD-10 (International Classification of Diseases 10th revision).

Statistical analysis

This study was reported following the STROBE statement. Absolute numbers or percentages were utilized to describe the distributions of maternal characteristics and maternal complications during pregnancy by the anemia status. We estimated the rate of each neonatal adverse outcome in women without anemia and those with mild, moderate and severe anemia. We did Mantel-Haenszel Chi-square test for trends for each neonatal outcome to compare trends in the rate of neonatal outcomes by different maternal anemia statuses. Binary logistic regression model was used to analyze the associations between neonatal outcomes and women with varying statuses of anemia during early pregnancy. Restricted cubic spline regression was conducted to address the potential nonlinearity of the association between maternal Hb levels and neonatal outcomes. Adjusted odds ratios (aOR) and 95% confidence intervals (95% CIs) were estimated by multivariate logistic regression to control for potential confounders. Restricted cubic spline regression also controlled the confounding factors. Sensitivity analyses were conducted by adjusting for different covariates in multivariate models. In model A, we adjusted for maternal age, maternal BMI, residence, education level, ethnicity, parity, folate use and per caput monthly family income. In model B, we additionally adjusted for complications during pregnancy including gestational diabetes mellitus, gestational hypertension, hyperlipidemia, hyperthyroidism, and placenta previa. EpiData version 3.1 (EpiData Association, Odense, Denmark) was used to develop the database by double entry to ensure the accuracy of the data. All analyses were performed using R software (version 3.5.0). All tests were two-tailed, with $P < 0.05$ set as the statistically significant difference.

RESULTS

Characteristics of Participants

We included 34,087 pregnant females in our study. A total of 5572 (16.3%) participants were diagnosed with anemia in the first trimester. The prevalence of mild anemia, moderate anemia and severe anemia was 10.9%, 5.2% and 0.3%, respectively. The participants were concentrated in the age group of <35 years (77.5%). 70.2% of the participants had a pre-pregnancy BMI of 18.5-23.9 kg/m^2, and most of the individuals lived in urban (61.8%). Their educational level was mostly senior middle school (28.4%) and college (45.7%). Of these individuals, 98.7% were of Han ethnicity, and 1.3% were members of other ethnic groups (includes>50 ethnic groups). Of the 34,087 pregnant women recruited in our study, a total of 15.9% of the participants reported gestational diabetes mellitus, 3.7% had gestational hypertension, and 1.8% reported with placenta

Confidential: For Review Only

previa. Distribution of maternal characteristics and complications by anemia statuses were displayed in Table 1.

Prevalence of neonatal adverse outcomes across maternal anemia status

The prevalence of preterm birth varied by maternal anemia status (Table 2), ranging from 10.9% among women without anemia to 40.0% among women with severe anemia (P_{trend}<0.001). Likewise, the prevalence of low birth weight and SGA infants raised from 7.6% to 44.0% and 14.1% to 35.0% among pregnant females with more severe anemia compared with no anemia, respectively (P_{trend}<0.001). There were positive relationships between the rate of preterm birth, low birth weight as well as SGA and the severity of maternal anemia. Whereas the statistically significant associations between the rate of congenital malformation and different maternal anemia statuses were not observed (P_{trend}>0.05).

Association of maternal anemia with the risk of neonatal outcomes

To assess the association between the severity of maternal anemia and adverse neonatal outcomes, we compared the risk of the neonatal outcomes for each anemia status to the risk of neonatal outcomes in women without anemia (Hb≥110 g/L). After adjusting for potential confounders including age, BMI, residence, education level, ethnicity, parity, folic acid use and per caput monthly family income (Table 3, model A), the risk estimates of preterm birth (mild anemia aOR 1.37 (95% CI 1.25-1.52), moderate anemia aOR 1.54 (95% CI 1.35-1.76), and severe anemia aOR 4.03 (95% CI 2.67-6.08), respectively), low birth weight (mild anemia aOR 1.61 (95% CI 1.44-1.79), moderate anemia aOR 2.01 (95% CI 1.75-2.30), and severe anemia aOR 6.11 (95% CI 3.99-9.36), respectively) and SGA (mild anemia aOR 1.37 (95% CI 1.25-1.52), moderate anemia aOR 1.54 (95% CI 1.35-1.76), and severe anemia aOR 2.61 (95% CI 1.74-4.50), respectively) significantly increased in offspring of pregnant females who had anemia. However, we did not find that women with anemia were associated with the risk of congenital malformation in offspring (mild anemia aOR 0.89 (95% CI 0.75-1.07), moderate anemia aOR 1.17 (95% CI 0.93-1.47), and severe anemia aOR 0.86 (95% CI 0.32-2.36), respectively). Figure 2 exhibited the restricted cubic spline for exploring the non-linear association between maternal Hb levels and the risk of neonatal outcomes after adjusting model A. Maternal Hb concentrations displayed the U-shaped relationship curve with the likelihood of preterm birth, low birth weight and SGA in offspring (all values $P_{non\text{-}linear}$<0.05). The significantly non-linear association between maternal Hb levels and the risk of congenital malformation in offspring was not observed ($P_{non\text{-}linear}$>0.05). The similar findings were illustrated in Figure 3 after adjusting model B. Adjusting for different covariates did not substantially affect the risk estimates of adverse neonatal outcomes, which were similar for model A and model B. AORs and 95% CIs for covariates in Model A and B associated with neonatal adverse outcomes were illustrated in Supplement Table 1.

DISCUSSION

We conducted a prospective cohort study using information about pregnant women in Hunan Provincial Maternal and Child Health Care Hospital, China. The prevalence of gestational anemia was 16.3% in the present study which was comparable with results previously reported in China[24, 25]. After adjusting for maternal confounders, maternal anemia during early pregnancy was associated with the risk of preterm birth, low birth weight and SGA. We also observed non-linear associations of maternal Hb concentrations with the risk of preterm birth, low birth weight and SGA in offspring. No association was found between anemia in early pregnancy and the risk

Confidential: For Review Only

of congenital malformations, nor was a non-linear association found between Hb levels in early pregnancy and the risk of congenital malformations. Sensitivity analysis verified the stability of the results. Furthermore, the positive association of the rate of preterm birth, low birth weight and SGA with the severity of maternal anemia in the first trimester was identified.

Published literature had already reported associations of anemia during pregnancy with neonatal outcomes such as preterm birth, low birth weight and SGA. However, these findings had not been consistent[26, 27, 28]. This might be that various researches defined anemia during pregnancy with different cutoff thresholds. Besides, the associations between maternal anemia and neonatal outcomes varied depending on the timing of the maternal Hb measurement[27]. In the current study, we specified that Hb was measured in the first trimester to analyze the associations between anemia during early pregnancy and neonatal outcomes. Our data showed that maternal anemia during early pregnancy had negative effects on preterm birth, low birth weight and SGA. Consistent with our findings, a meta-analysis reported an increased risk of low birth weight among women with lower Hb concentrations during overall or any stage of pregnancy[8]. Associations between maternal anemia in the first trimester and neonatal outcomes were inconclusive. According to the results of Ronkainen *et al.*,[28] the risk of SGA increased in the offspring of pregnant females whose level of Hb was <11.0 g/dl in the first trimester. But this study showed that no association between low maternal Hb level and preterm birth. A study performed in the UK came to the same conclusion that low maternal Hb during early pregnancy is associated with SGA, but not preterm birth[29]. However, Ren's result implied that rates of low birth weight, preterm birth, and SGA increased with the decrease in first-trimester Hb concentration in this Chinese population which was consistent with our research results[30]. Additionally, our study discovered that maternal Hb concentrations displayed a U-shaped relationship with the risk of preterm birth, low birth weight and SGA in offspring. Consistent with our findings, a study conducted in the UK reported a U-shaped association between maternal Hb values and the risk of low birth weight and preterm birth[31]. A study performed in Chinese pregnant females indicated that U-shaped relationships between first-trimester Hb concentrations and the risk of preterm birth, low birth weight, and SGA were observed without adjusting for confounding factors[32].

Hb carries oxygen to the placenta, which is essential for normal fetal growth in the uterus. Anemia or low Hb levels may stimulate changes in placental angiogenesis and affect placental transit function[33]. As a result, it reduces the oxygen supply and nutrient supplementation to the fetus, resulting in intrauterine growth retardation and low birth weight[34]. Increased placental vascularization in women with low Hb levels suggests earlier placental maturation, which may contribute to an increased risk of preterm birth[34]. Our study discovered that pregnant females with underweight were associated with severe anemia. An Indian cohort study suggested that over third anemic pregnant women coexisted with underweight, and were associated with the increased risk of low birth weight in offspring[35], implying that maternal anemia possibly caused by malnutrition statuses had an impact on intrauterine fetal nutrition and led to low birth weight in the offspring. In addition, low Hb may lead to chronic hypoxia which in turn may cause a stress response of increased placental corticotropin-releasing hormone secretion, inducing adverse consequences such as preterm birth, gestational hypertension and so forth.[36] Nevertheless, the underlying biological mechanisms involved in the associations between maternal anemia and preterm birth, low birth weight as well as SGA remain to be further elucidated.

Organogenesis occurs in the first trimester of pregnancy, during which the primary structures of

Confidential: For Review Only

most organs develop such as the heart, lung, liver, and so on. In the early stage of development, the embryo and the fetus undergo intense epigenetic reprogramming[14, 15]. Previous studies have shown that epigenetic changes might play a significant role in offspring diseases caused by gestational complications[16]. Gestational anemia is one of the most common gestational complications. Our study revealed lack of association between maternal anemia during early pregnancy and congenital malformation. Shi *et al.*[37] also found that no association between low birth weight and maternal anemia during overall pregnancy.

Significantly, our findings suggested that pregnant females with severe anemia had a lower prevalence of gestational diabetes mellitus and Lin *et al.*[38] also provided evidence that the prevalence of gestational diabetes mellitus decreased in women with anemia. Additionally, Lao *et al.*[39] discovered the association of iron deficiency anemia with the decreased prevalence of gestational diabetes mellitus. Conversely, Liu *et al.*[40] yielded a different result that there was no significant association between iron deficiency anemia status and GDM risk. Therefore, the association between anemia and gestational diabetes mellitus remains to be further explored.

This study utilized a large sample of pregnant women and took place in a prospective cohort study which minimized recall bias and ensured the reliability of the study. Additionally, our study specified that Hb was measured during early pregnancy to analyze the association between anemia during early pregnancy and neonatal outcomes. However, some limitations should be considered. Firstly, the study objects were all from the same hospital, and the sample sources may be concentrated in a certain group of population. This may affect the representativeness of the sample and thus affect extrapolation. Secondly, we did not collect information on maternal anemia remedies and Hb concentrations in childbirth. Thirdly, there was no assurance that the results would not be impacted by residual confounding factors even after we adjusted for a large number of potential confounding factors.

CONCLUSION

In conclusion, it is of concern that anemia during early pregnancy regardless of the severity may significantly increase the risk of neonatal outcomes such as preterm birth, low birth weight and SGA. According to the results of our study, we suggest that the rate of neonatal outcomes may increase with the severity of maternal anemia. However, more prospective studies with larger samples are needed to verify the authenticity of the results and explore the underlying mechanisms. Pre-pregnancy screening for anemia and implementation of interventions are necessary to ensure adequate Hb concentrations during pregnancy and to reduce the adverse effects of maternal anemia on the health of their offspring.

Funding

This work was supported by the Project Funded by National Natural Science Foundation Program of China (82073653 and 81803313), Hunan Outstanding Youth Fund Project (2022JJ10087), Hunan Provincial Science and Technology Talent Support Project (2020TJ-N07), Hunan Provincial Key Research and Development Program (2018SK2063), Open Project from NHC Key Laboratory of Birth Defect for Research and Prevention (KF2020006), Natural Science Foundation of Hunan Province (2018JJ2551) and Science and Technology Planning Project of Guangdong Province (2020A1414010152).

Confidential: For Review Only

Author Contributions

YPL and TWZ wrote the main manuscript text; YGC and TWZ analyzed the data statistical analyses; JBQ, YGC and YPL reviewed and revised the manuscript; XYL, YPL, XLS, SMZ, MTS, JHW and JS have collected the data. All authors have read and approved the final version of the manuscript.

Patient and Public Involvement Statement

Patients or the public were involved in the design, or conduct, or reporting, or dissemination plans of our research. Refer to the Methods section for further details.

Patient consent for publication

Not required.

Ethics approval

This study was approved by the ethics committee of the Xiangya School of Public Health of Central South University, and registered in the Chinese Clinical Trial Registry (Registration number: ChiCTR1800016635).

Provenance and peer review

Not commissioned; externally peer reviewed.

Data Availability Statement

The datasets used and/or analyzed during the current study are available from the corresponding author on reasonable request.

Acknowledgments

The authors would like to thank the editors and reviewers for their suggestions and all colleagues working in Maternal and Child Health Promotion and Birth Defect Prevention Group.

Conflict of Interest

The authors declared no potential conflicts of interest with respect to the research, authorship, and/or publication of this article.

Reference

1. Balarajan, Y.; Ramakrishnan, U.; Ozaltin, E.; Shankar, A.H.; Subramanian, S.V. Anaemia in low-income and middle-income countries. *Lancet*. **2011**, *378*, 2123-2135.
2. McLean, E.; Cogswell, M.; Egli, I.; Wojdyla, D.; de Benoist, B. Worldwide prevalence of anaemia, WHO Vitamin and Mineral Nutrition Information System, 1993-2005. *Public Health Nutr*. **2009**, *12*, 444-454.
3. Black, R.E.; Victora, C.G.; Walker, S.P.; Bhutta, Z.A.; Christian, P.; de Onis, M.; Ezzati, M.; Grantham-McGregor, S.; Katz, J.; Martorell, R.; Uauy, R. Maternal and child undernutrition and overweight in low-income and middle-income countries. *Lancet*. **2013**, *382*, 427-451.
4. Stevens, G.A.; Finucane, M.M.; De-Regil, L.M.; Paciorek, C.J.; Flaxman, S.R.; Branca, F.; Peña-Rosas, J.P.; Bhutta, Z.A.; Ezzati, M. Global, regional, and national trends in haemoglobin concentration and prevalence of total and severe anaemia in children and pregnant and non-pregnant women for 1995-2011: a systematic analysis of population-representative data. *Lancet Glob. Health*. **2013**, *1*, e16-e25.
5. Organization, N.; Health, W. Global nutrition targets 2025: Anaemia policy brief. *Working Papers*. **2016**.
6. Haider, B.A.; Olofin, I.; Wang, M.; Spiegelman, D.; Ezzati, M.; Fawzi, W.W. Anaemia, prenatal iron use, and risk of adverse pregnancy outcomes: systematic review and meta-analysis. *BMJ-*

Confidential: For Review Only

British Medical Journal. **2013**, *346*, f3443.

7. Jung, J.; Rahman, M.M.; Rahman, M.S.; Swe, K.T.; Islam, M.R.; Rahman, M.O.; Akter, S. Effects of hemoglobin levels during pregnancy on adverse maternal and infant outcomes: a systematic review and meta-analysis. *Ann. N.Y. Acad. Sci.* **2019**, *1450*, 69-82.
8. Young, M.F.; Oaks, B.M.; Tandon, S.; Martorell, R.; Dewey, K.G.; Wendt, A.S. Maternal hemoglobin concentrations across pregnancy and maternal and child health: a systematic review and meta-analysis. *Ann. N.Y. Acad. Sci.* **2019**, *1450*, 47-68.
9. Malhotra, M.; Sharma, J.B.; Batra, S.; Sharma, S.; Murthy, N.S.; Arora, R. Maternal and perinatal outcome in varying degrees of anemia. *Int. J. Gynecol. Obstet.* **2002**, *79*, 93-100.
10. Bora, R.; Sable, C.; Wolfson, J.; Boro, K.; Rao, R. Prevalence of anemia in pregnant women and its effect on neonatal outcomes in Northeast India. *J. Matern.-Fetal Neonatal Med.* **2014**, *27*, 887-891.
11. Menon, K.C.; Ferguson, E.L.; Thomson, C.D.; Gray, A.R.; Zodpey, S.; Saraf, A.; Das, P.K.; Skeaff, S.A. Effects of anemia at different stages of gestation on infant outcomes. *Nutrition*. **2016**, *32*, 61-65.
12. de Haas, S.; Ghossein-Doha, C.; van Kuijk, S.M.; van Drongelen, J.; Spaanderman, M.E. Physiological adaptation of maternal plasma volume during pregnancy: a systematic review and meta-analysis. *Ultrasound Obstet. Gynecol.* **2017**, *49*, 177-187.
13. Díaz-López, A.; Ribot, B.; Basora, J.; Arija, V. High and Low Haemoglobin Levels in Early Pregnancy Are Associated to a Higher Risk of Miscarriage: A Population-Based Cohort Study. *Nutrients*. **2021**, *13*.
14. Liu, X.; Wang, C.; Liu, W.; Li, J.; Li, C.; Kou, X.; Chen, J.; Zhao, Y.; Gao, H.; Wang, H.; Zhang, Y.; Gao, Y.; Gao, S. Distinct features of H3K4me3 and H3K27me3 chromatin domains in pre-implantation embryos. *Nature*. **2016**, *537*, 558-562.
15. Wang, C.; Liu, X.; Gao, Y.; Yang, L.; Li, C.; Liu, W.; Chen, C.; Kou, X.; Zhao, Y.; Chen, J.; Wang, Y.; Le R; Wang, H.; Duan, T.; Zhang, Y.; Gao, S. Reprogramming of H3K9me3-dependent heterochromatin during mammalian embryo development. *Nat. Cell Biol.* **2018**, *20*, 620-631.
16. Sun, C.F.; Liu, H.; Hao, Y.H.; Hu, H.T.; Zhou, Z.Y.; Zou, K.X.; Liu, X.M.; Sheng, J.Z.; Ding, G.L.; Huang, H.F. Association between gestational anemia in different trimesters and neonatal outcomes: a retrospective longitudinal cohort study. *World Journal of Pediatrics*. **2021**, *17*, 197-204.
17. Hämäläinen, H.; Hakkarainen, K.; Heinonen, S. Anaemia in the first but not in the second or third trimester is a risk factor for low birth weight. *Clin. Nutr.* **2003**, *22*, 271-275.
18. Organization, W.H. EN VMNIS - Indicators - Haemoglobin concentrations for the diagnosis of anaemia and assessment of severity. **2011**.
19. Ananth, C.V.; VanderWeele, T.J. Placental abruption and perinatal mortality with preterm delivery as a mediator: disentangling direct and indirect effects. *Am. J. Epidemiol.* **2011**, *174*, 99-108.
20. Fadl, N.; Ice, G.H.; Haile, Z.T. Association between maternal high-risk factors and anemia among women in Ethiopia. *Nutrition*. **2021**, *91-92*, 111404.
21. Sharma, D.; Amgain, K.; Panta, P.P.; Pokhrel, B. Hemoglobin levels and anemia evaluation among pregnant women in the remote and rural high lands of mid-western Nepal: a hospital based study. *BMC Pregnancy Childbirth*. **2020**, *20*, 182.

Confidential: For Review Only

22. Churchill, D.; Nair, M.; Stanworth, S.J.; Knight, M. The change in haemoglobin concentration between the first and third trimesters of pregnancy: a population study. *BMC Pregnancy Childbirth*. **2019**, *19*, 359.
23. Qu, Y.; Lin, S.; Zhuang, J.; Bloom, M.S.; Smith, M.; Nie, Z.; Mai, J.; Ou, Y.; Wu, Y.; Gao, X.; Tan, H.; Liu, X. First-Trimester Maternal Folic Acid Supplementation Reduced Risks of Severe and Most Congenital Heart Diseases in Offspring: A Large Case-Control Study. *J. Am. Heart Assoc*. **2020**, *9*, e15652.
24. Tan, J.; He, G.; Qi, Y.; Yang, H.; Xiong, Y.; Liu, C.; Wang, W.; Zou, K.; Lee, A.H.; Sun, X.; Liu, X. Prevalence of anemia and iron deficiency anemia in Chinese pregnant women (IRON WOMEN): a national cross-sectional survey. *BMC Pregnancy Childbirth*. **2020**, *20*, 670.
25. Wu, Y.; Ye, H.; Liu, J.; Ma, Q.; Yuan, Y.; Pang, Q.; Liu, J.; Kong, C.; Liu, M. Prevalence of anemia and sociodemographic characteristics among pregnant and non-pregnant women in southwest China: a longitudinal observational study. *BMC Pregnancy Childbirth*. **2020**, *20*, 535.
26. Zhang, X.; Xu, Q.; Yang, Y.; Wang, L.; Liu, F.; Li, Q.; Ji, M.; He, Y.; Wang, Y.; Zhang, Y.; Zhang, H.; Peng, Z.; Ma, X.; Yu, Z. Preconception Hb concentration and risk of preterm birth in over 2·7 million Chinese women aged 20-49 years: a population-based cohort study. *Br. J. Nutr.* **2018**, *120*, 508-516.
27. Liu, D.; Li, S.; Zhang, B.; Kang, Y.; Cheng, Y.; Zeng, L.; Chen, F.; Mi, B.; Qu, P.; Zhao, D.; Zhu, Z.; Yan, H.; Wang, D.; Dang, S. Maternal Hemoglobin Concentrations and Birth Weight, Low Birth Weight (LBW), and Small for Gestational Age (SGA): Findings from a Prospective Study in Northwest China. *Nutrients*. **2022**, *14*.
28. Ronkainen, J.; Lowry, E.; Heiskala, A.; Uusitalo, I.; Koivunen, P.; Kajantie, E.; Vääräsmäki, M.; Järvelin, M.R.; Sebert, S. Maternal hemoglobin associates with preterm delivery and small for gestational age in two Finnish birth cohorts. *Eur. J. Obstet. Gynecol. Reprod. Biol.* **2019**, *238*, 44-48.
29. Alwan, N.A.; Cade, J.E.; McArdle, H.J.; Greenwood, D.C.; Hayes, H.E.; Simpson, N.A. Maternal iron status in early pregnancy and birth outcomes: insights from the Baby's Vascular health and Iron in Pregnancy study. *Br. J. Nutr.* **2015**, *113*, 1985-1992.
30. Ren, A.; Wang, J.; Ye, R.W.; Li, S.; Liu, J.M.; Li, Z. Low first-trimester hemoglobin and low birth weight, preterm birth and small for gestational age newborns. *Int. J. Gynecol. Obstet.* **2007**, *98*, 124-128.
31. Steer, P.; Alam, M.A.; Wadsworth, J.; Welch, A. Relation between maternal haemoglobin concentration and birth weight in different ethnic groups. *BMJ-British Medical Journal*. **1995**, *310*, 489-491.
32. Zhou, L.M.; Yang, W.W.; Hua, J.Z.; Deng, C.Q.; Tao, X.; Stoltzfus, R.J. Relation of hemoglobin measured at different times in pregnancy to preterm birth and low birth weight in Shanghai, China. *Am. J. Epidemiol.* **1998**, *148*, 998-1006.
33. Figueiredo, A.; Gomes-Filho, I.S.; Batista, J.; Orrico, G.S.; Porto, E.; Cruz, P.R.; Dos, S.C.S.; Brito, S.M.; Ramos, M.; Sena, M.; Vilasboas, S.; Seixas, D.C.S.; Pereira, M.G. Maternal anemia and birth weight: A prospective cohort study. *Plos One*. **2019**, *14*, e212817.
34. Stangret, A.; Wnuk, A.; Szewczyk, G.; Pyzlak, M.; Szukiewicz, D. Maternal hemoglobin concentration and hematocrit values may affect fetus development by influencing placental angiogenesis. *J. Matern.-Fetal Neonatal Med.* **2017**, *30*, 199-204.
35. Patel, A.; Prakash, A.A.; Das, P.K.; Gupta, S.; Pusdekar, Y.V.; Hibberd, P.L. Maternal anemia

Confidential: For Review Only

and underweight as determinants of pregnancy outcomes: cohort study in eastern rural Maharashtra, India. *BMJ Open*. **2018**, *8*, e21623.

36. Bakacak, M.; Avci, F.; Ercan, O.; Köstü, B.; Serin, S.; Kiran, G.; Bostanci, M.S.; Bakacak, Z. The effect of maternal hemoglobin concentration on fetal birth weight according to trimesters. *J. Matern.-Fetal Neonatal Med.* **2015**, *28*, 2106-2110.
37. Shi, H.; Chen, L.; Wang, Y.; Sun, M.; Guo, Y.; Ma, S.; Wang, X.; Jiang, H.; Wang, X.; Lu, J.; Ge, L.; Dong, S.; Zhuang, Y.; Zhao, Y.; Wei, Y.; Ma, X.; Qiao, J. Severity of Anemia During Pregnancy and Adverse Maternal and Fetal Outcomes. *Jama Netw. Open*. **2022**, *5*, e2147046.
38. Lin, L.; Wei, Y.; Zhu, W.; Wang, C.; Su, R.; Feng, H.; Yang, H. Prevalence, risk factors and associated adverse pregnancy outcomes of anaemia in Chinese pregnant women: a multicentre retrospective study. *BMC Pregnancy Childbirth*. **2018**, *18*, 111.
39. Lao, T.T.; Ho, L.F. Impact of iron deficiency anemia on prevalence of gestational diabetes mellitus. *Diabetes Care*. **2004**, *27*, 650-656.
40. Liu, Q.; Wei, S.; Wang, F. Prothrombotic state and calcium deficiency in early pregnancy are risk factors for gestational diabetes mellitus: a retrospective cohort study. *Gynecol. Endocrinol.* **2022**, *38*, 407-410.

Figure legend:

Figure 1: Flow chart showing the process of participant's recruitment

Figure 2: Restricted cubic spline of the association between maternal Hb levels and the risk of neonatal outcomes after adjusting model A. Model A adjusted for maternal age, maternal BMI, residence, education level, ethnicity, parity, folate use, and per caput monthly family income. SGA= small for gestational age; OR=odds ratio; 95% CI = 95% confidence interval

Figure 3: Restricted cubic spline of the association between maternal Hb levels and the risk of neonatal outcomes after adjusting model B. Model B adjusted for all maternal complications during pregnancy in addition to covariates in model A. SGA= small for gestational age; OR=odds ratio; 95% CI = 95% confidence interval

Confidential: For Review Only

Table 1 Distribution of maternal characteristics and complications by anemia status

Characteristics	Total<br>*N* (%)	Maternal hemoglobin, *N* (%)			
		Normal<br>≥110 g/L	Mild<br>100-109 g/L	Moderate<br>70–99 g/L	Severe<br><70 g/L
Deliveries	34087	28515 (83.7)	3703 (10.9)	1769 (5.2)	100 (0.3)
Maternal age (years)					
<35	26433 (77.5)	22082 (77.4)	2890 (78.0)	1382 (78.1)	79 (79.0)
≥35	7654 (22.5)	6433 (22.6)	813 (22.0)	387 (21.9)	21 (21.0)
Maternal BMI					
<18.5	4920 (14.4)	3978 (14.0)	574 (15.5)	336 (19.0)	32 (32.0)
18.5-23.9	23913 (70.2)	20123 (70.6)	2547 (68.8)	1184(76.9)	59 (59.0)
24-27.9	4329(12.7)	3653 (12.8)	472 (12.7)	195 (11.0)	9 (9.0)
≥28	925 (2.7)	761 (2.7)	110 (3.0)	54 (3.1)	0 (0.0)
Residence					
Urban	21062 (61.8)	17672 (62.0)	2253 (60.8)	1082 (61.2)	55 (55.0)
Rural	13025 (38.2)	10843 (38.0)	1450 (39.2)	687 (38.8)	45 (45.0)
Education level					
Junior high school or below	2547 (7.5)	1705 (6.0)	516 (13.9)	299 (16.9)	27 (27.0)
Senior middle school	9684 (28.4)	7842 (27.5)	1218 (32.9)	594 (33.6)	30 (30.0)
College	15588 (45.7)	13618 (47.8)	1346 (36.3)	586 (33.1)	38 (38.0)
Master or above	6268 (18.4)	5350 (18.8)	623 (18.8)	290 (16.4)	5 (5.0)
Ethnicity					
Han	33639 (98.7)	28129 (98.6)	3686 (99.5)	1730 (97.8)	94 (94.0)
Minority	448 (1.3)	386 (1.4)	17 (0.5)	39 (2.2)	6 (6.0)
Parity					
0	10488 (30.8)	9274 (32.5)	813 (22.0)	369 (20.9)	32 (32.0)
1-3	20189 (59.2)	16601 (58.2)	2362 (62.8)	1168 (66.0)	58 (58.0)
≥4	3410 (10.0)	2640 (9.3)	528 (14.3)	232 (13.1)	10 (10.0)
Per caput monthly family income (RMB)					
≤2500	5875(17.2)	4916(17.2)	622(16.8)	322(18.2)	15(15.0)
2500-5000	18206(53.4)	15225(53.4)	1980(53.5)	944(53.4)	57(57.0)
>5000	10006(29.4)	8374(29.4)	1101(29.7)	503(28.4)	28(28.0)
Folic acid use *	32540 (95.5)	27272 (95.6)	3490 (94.2)	1687 (95.4)	91 (91.0)
Gestational diabetes mellitus	5424 (15.9)	4689 (16.4)	485 (13.1)	250 (14.1)	0 (0.0)
Gestational hypertension	1246 (3.7)	1012 (3.5)	161 (4.3)	68 (3.8)	5 (5.0)
Hyperlipidemia	375 (1.1)	312 (1.1)	35 (0.9)	28 (1.6)	0 (0.0)
Hyperthyroidism	636 (1.9)	542 (1.9)	71 (1.9)	23 (1.3)	0 (0.0)
Placenta previa	606 (1.8)	492 (1.7)	70 (1.9)	42 (2.4)	2 (2.0)

BMI = body mass index

Confidential: For Review Only

* Folic acid use before or during pregnancy

Table 2 Prevalence (% and 95% CI) of neonatal adverse outcomes according to maternal anemia status

Maternal anemia	Preterm birth % (95% CI)	Low birth weight % (95% CI)	SGA % (95% CI)	Congenital malformation % (95% CI)
Normal (≥110 g/L)	10.9 (10.5-11.2)	7.6 (7.2-7.9)	14.1 (13.7-14.5)	4.4 (4.2-4.7)
Mild (100-109 g/L)	15.7 (14.5-16.9)	13.7 (12.6-14.8)	20.2 (18.9-21.5)	3.9 (3.3-4.6)
Moderate (70–99 g/L)	18.1 (16.3-19.9)	17.8 (16.0-19.6)	22.8 (20.8-24.7)	5.0 (4.0-6.0)
Severe (<70 g/L)	40.0 (30.2-49.8)	44.0 (34.1-53.9)	35.0 (25.5-44.5)	4.0 (0.1-7.9)
P_{trend}	<0.001	<0.001	<0.001	0.899

SGA= small for gestational age; 95% CI = 95% confidence interval.

Confidential: For Review Only

1 Table 3 Adjusted risk ratios for neonatal adverse outcomes associated with severity of anemia during early pregnancy [a]

Outcome	Model A[b]				Model B [c]			
	Mild 100-109 g/L	Moderate 70–99 g/L	Severe <70 g/L	Overall <110 g/L	Mild 100-109 g/L	Moderate 70–99 g/L	Severe <70 g/L	Overall <110 g/L
Preterm birth	1.37(1.25-1.52) *	1.54(1.35-1.76) *	4.03(2.67-6.08) *	1.47(1.35-1.59) *	1.37(1.24-1.51) *	1.55(1.36-1.77) *	4.05(2.68-6.12) *	1.46(1.35-1.59) *
Low birth weight	1.61(1.44-1.79) *	2.01(1.75-2.30) *	6.11(3.99-9.36) *	1.80(1.64-1.96) *	1.60(1.43-1.78) *	2.02(1.76-2.32) *	6.25(4.06-9.61) *	1.79(1.64-1.96) *
SGA	1.37(1.25-1.52) *	1.54(1.35-1.76) *	2.61(1.74-4.50) *	1.47(1.35-1.59) *	1.37(1.24-1.51) *	1.55(1.36-1.77) *	2.60(1.73-4.49) *	1.46(1.35-1.59) *
Congenital malformation	0.89(0.75-1.07)	1.17(0.93-1.47)	0.86(0.32-2.36)	0.98(0.85-1.13)	0.88(0.74-1.06)	1.15(0.92-1.44)	0.83(0.30-2.28)	0.96(0.83-1.12)

2 SGA= small for gestational age.

3 [a] Non-anemic pregnant women as reference.

4 [b] Model A adjusted for maternal age, maternal BMI, residence, education level, ethnicity, parity, folate use, and per caput monthly family income.

5 [c] Model B adjusted for all maternal complications during pregnancy in addition to covariates in model A.

6 * $P < 0.005$

Confidential: For Review Only

Confidential: For Review Only

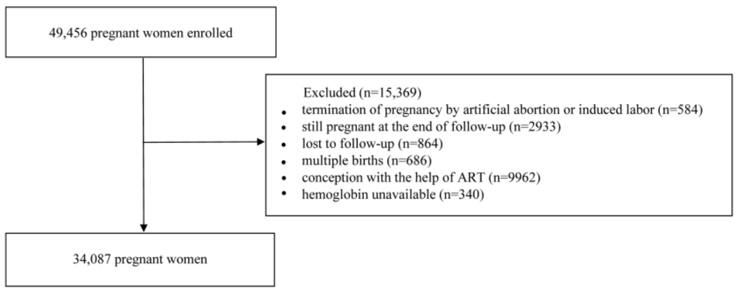

Flow chart showing the process of participant's recruitment

279x215mm (300 x 300 DPI)

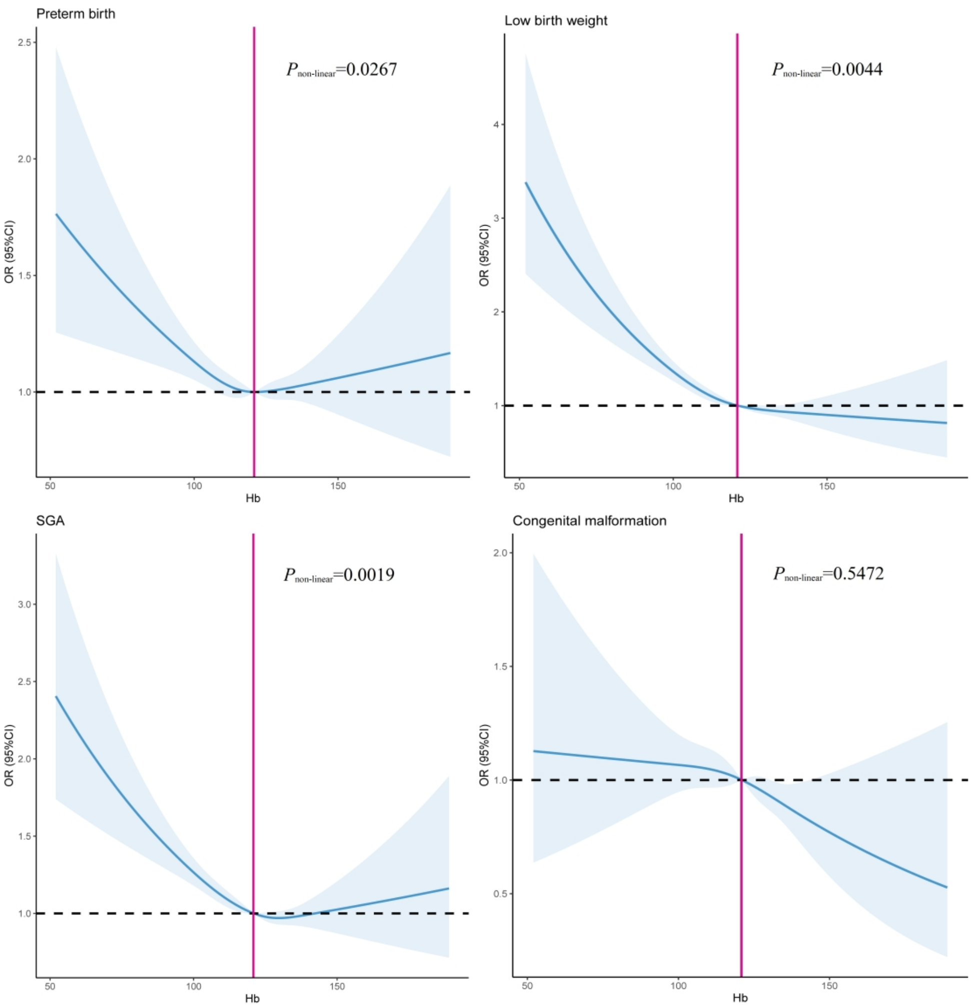

Restricted cubic spline of the association between maternal Hb levels and the risk of neonatal outcomes after adjusting model A. Model A adjusted for maternal age, maternal BMI, residence, education level, ethnicity, parity, folate use, and per caput monthly family income. SGA= small for gestational age; OR=odds ratio; 95% CI = 95% confidence interval

908x938mm (168 x 168 DPI)

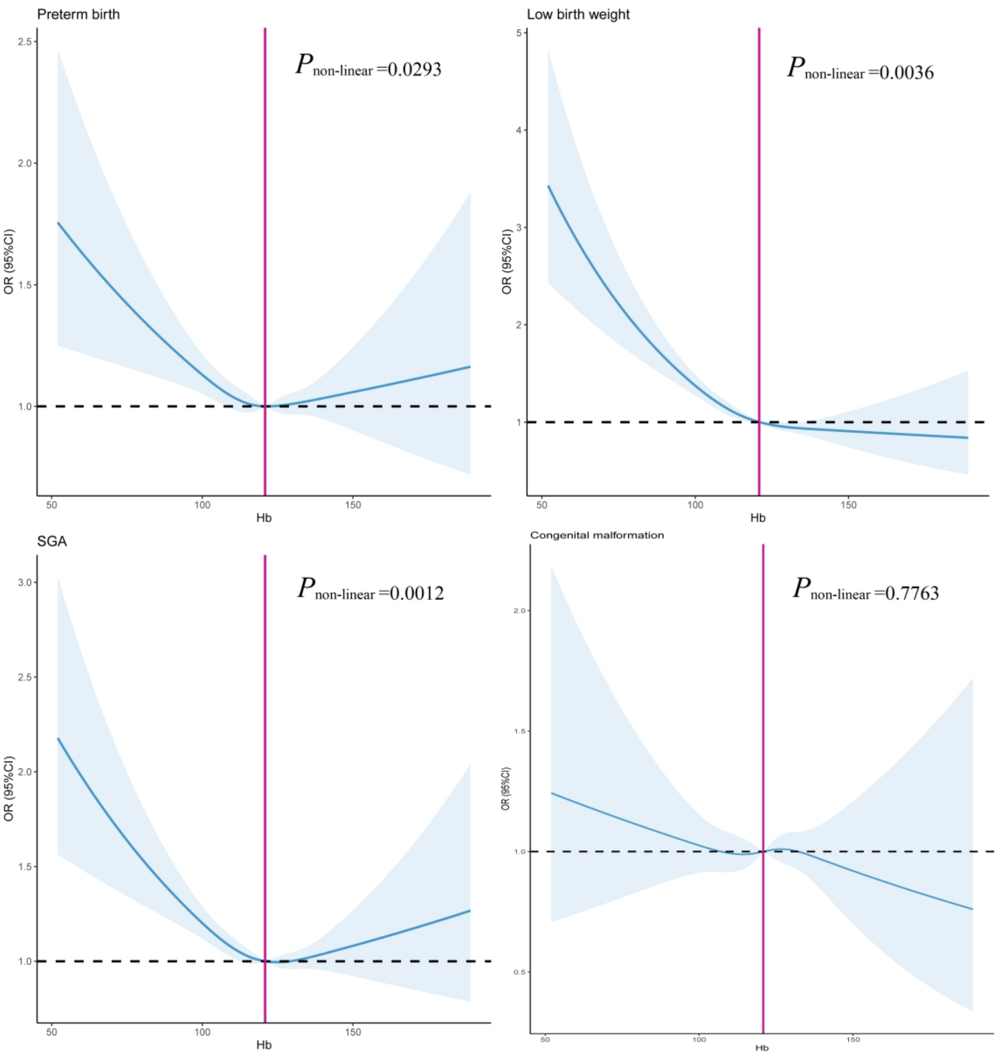

Restricted cubic spline of the association between maternal Hb levels and the risk of neonatal outcomes after adjusting model B. Model B adjusted for all maternal complications during pregnancy in addition to covariates in model A. SGA= small for gestational age; OR=odds ratio; 95% CI = 95% confidence interval

323x340mm (300 x 300 DPI)

Supplement Table 1 Adjusted risk ratios for covariates in Model A and B associated with neonatal adverse outcomes

Covariates	Model A[b]				Model B[c]			
	Preterm birth	Low birth weight	SGA	Congenital malformation	Preterm birth	Low birth weight	SGA	Congenital malformation
Maternal age (years)								
<35	Reference	Reference	Reference	Reference	Reference	Reference	Reference	Reference
≥35	1.07(0.99-1.16)	1.37(1.23-1.51) *	1.17(1.06-1.28) *	1.11(0.95-1.29) *	1.07(0.99-1.16)	1.36(1.23-1.52) *	1.16(1.06-1.28) *	1.11(0.96-1.29) *
Maternal BMI								
18.5-23.9	Reference	Reference	Reference	Reference	Reference	Reference	Reference	Reference
<18.5	2.08(1.20-3.60) *	4.19(2.34-7.49) *	2.35(1.30-4.27) *	-	2.04(1.18-3.53) *	3.97(2.21-7.13) *	2.29(1.26-4.15) *	-
24-27.9	0.79(0.74-0.85) *	0.51(0.46-0.56) *	0.73(0.67-0.79) *	1.17(1.00-1.37) *	0.79(0.74-0.85) *	0.50(0.45-0.55) *	0.73(0.67-0.79) *	1.17(1.01-1.37) *
≥28	0.75(0.69-0.81) *	0.46(0.41-0.51) *	0.65(0.59-0.72) *	1.43(1.22-1.51) *	0.75(0.70-0.82) *	0.46(0.41-0.51) *	0.66(0.60-0.73) *	1.43(1.21-1.67) *
Residence								
Urban	Reference	Reference	Reference	Reference	Reference	Reference	Reference	Reference
Rural	1.22(1.16-1.29) *	1.97(1.82-2.13) *	1.40(1.30-1.50) *	1.36(1.22-1.51) *	1.23(1.16-1.29) *	2.01(1.85-2.17) *	1.40(1.31-1.51) *	1.32(1.19-1.47) *
Education level								
Junior high school or below	Reference	Reference	Reference	Reference	Reference	Reference	Reference	Reference
Senior middle school	0.74(0.66-0.84) *	0.60(0.61-0.70) *	0.70(0.61-0.81) *	0.89(0.68-1.15)	0.74(0.66-0.84) *	0.59(0.51-0.69) *	0.70(0.61-0.81) *	0.87(0.67-1.14)
College	0.61(0.56-0.67) *	0.34(0.30-0.38) *	0.52(0.47-0.58) *	0.78(0.65-0.95) *	0.61(0.56-0.67) *	0.33(0.29-0.37) *	0.52(0.47-0.58) *	0.78(0.64-0.95) *
Master or above	0.53(0.47-0.60) *	0.20(0.17-0.24) *	0.40(0.35-0.47) *	0.89(0.70-1.12)	0.53(0.47-0.59) *	0.19(0.16-0.24) *	0.41(0.34-0.47) *	0.87(0.69-1.11)
Ethnicity								
Han	Reference	Reference	Reference	Reference	Reference	Reference	Reference	Reference

Confidential: For Review Only

Minority	0.83(0.66-1.04)	0.81(0.58-1.13)	0.86(0.65-1.14)	0.72(0.48-1.06)	0.82(0.66-1.03)	0.78(0.56-1.10)	0.85(0.63-1.13)	0.72(0.49-1.08)
Parity								
0	Reference	Reference	Reference	Reference	Reference	Reference	Reference	Reference
1-3	1.01(0.95-1.08)	1.17(1.06-1.29) *	1.04(0.96-1.13)	0.84(0.75-0.95) *	1.01(0.95-1.08)	1.18(1.07-1.29) *	1.04(0.96-1.12)	0.85(0.75-0.95) *
≥4	1.09(0.98-1.21)	1.43(1.24-1.65) *	1.13(0.99-1.29)	0.73(0.59-0.91) *	1.09(0.98-1.21)	1.41(1.22-1.64) *	1.13(0.98-1.28)	0.73(0.59-0.91) *
Per caput monthly family income (RMB)								
≤2500	Reference	Reference	Reference	Reference	Reference	Reference	Reference	Reference
2500-5000	1.08(1.00-1.16) *	1.27(1.13-1.42) *	1.17(1.06-1.28) *	0.84(0.73-0.97) *	1.09(1.01-1.18) *	1.33(1.18-1.49) *	1.19(1.08-1.31) *	0.86(0.74-0.99) *
>5000	0.95(0.87-1.03)	0.92(0.81-1.05)	0.98(0.89-1.10)	0.88(0.75-1.02)	0.96(0.88-1.04)	0.94(0.83-1.08)	0.99(0.89-1.11)	0.92(0.78-1.05)
Folic acid use *								
Yes	Reference	Reference	Reference	Reference	Reference	Reference	Reference	Reference
No	1.21(1.07-1.36) *	1.76(1.52-2.05) *	1.35(1.17-1.56) *	1.57(1.28-1.94) *	1.21(1.07-1.36) *	1.78(1.53-2.08) *	1.35(1.17-1.56) *	1.58(1.28-1.95) *
Gestational diabetes mellitus								
Yes	-	-	-	-	Reference	Reference	Reference	Reference
No	-	-	-	-	0.97(0.91-1.05)	1.10(0.99-1.23)	1.02(0.93-1.11)	0.87(0.75-1.02)
Gestational hypertension								
Yes	-	-	-	-	Reference	Reference	Reference	Reference
No	-	-	-	-	1.82(1.60-2.06) *	3.95(3.42-4.56) *	2.26(1.97-2.61) *	0.88(0.65-1.19)
Hyperlipidemia								
Yes	-	-	-	-	Reference	Reference	Reference	Reference
No	-	-	-	-	1.05(0.82-1.35)	1.06(0.74-1.53)	1.14(0.84-1.55)	0.86(0.50-1.47)

Confidential: For Review Only

Hyperthyroidism								
Yes	-	-	-	-	Reference	Reference	Reference	Reference
No	-	-	-	-	0.97(0.77-1.18)	0.46(0.32-0.67) *	0.99(0.78-1.27)	0.86(0.57-1.30)
Placenta previa								
Yes	-	-	-	-	Reference	Reference	Reference	Reference
No	-	-	-	-	0.81(0.65-1.01)	0.53(0.37-0.76) *	0.67(0.50-0.89) *	3.91(3.09-4.93) *

SGA= small for gestational age.

[b] Model A adjusted for maternal age, maternal BMI, residence, education level, ethnicity, parity, folate use, and per caput monthly family income.

[c] Model B adjusted for all maternal complications during pregnancy in addition to covariates in model A.

* $P < 0.005$.

Confidential: For Review Only
